# Oxygen-dependent subcellular redistribution of PHD3 links the hypoxic microenvironment to mitochondrial metabolic reprogramming in ccRCC

**DOI:** 10.1038/s12276-026-01814-z

**Published:** 2026-09-02

**Authors:** Jaelim Sim, Soyeon Lim, Changjun Lee, Hani Jieun Kim, Wonseok Lee, Hyeonki Kim, Hojun Lee, Kimyeong Kim, Mahesh Kumar Teli, Kyeong Beom Jo, Jacob Wright, Jee Soo Park, Myung Eun Lee, Minsun Jung, Minsoo Noh, Dae-Kyum Kim, Mi-Hyun Kim, Dong Wook Choi, Tae Su Choi, Won Sik Ham, Hunsang Lee, Haejin Yoon

**Affiliations:** 1 Department of Life Sciences, College of Life Sciences and Biotechnology, Korea University, Seoul, Republic of Korea; 2 Department of Biological Sciences, Ulsan National Institute of Science and Technology, Ulsan, Republic of Korea; 3 The Kinghorn Cancer Centre and Cancer Research Theme, Garvan Institute of Medical Research, Darlinghurst, New South Wales Australia; 4 St Vincent’s Clinical School, Faculty of Medicine, University of New South Wales, Sydney, New South Wales Australia; 5 National Research Laboratory for Convergence Degradation Biology, Korea University, Seoul, South Korea; 6 Department of Biotechnology, College of Life Sciences and Biotechnology, Korea University, Seoul, Republic of Korea; 7 Gachon Institute of Pharmaceutical Science & Department of Pharmacy, College of Pharmacy, Gachon University, Incheon, Republic of Korea; 8 Division of Thoracic and Upper Gastrointestinal Surgery, Department of Surgery, Faculty of Medicine and Health Sciences, McGill University, Montreal, Quebec, Canada; 9 Cancer Research Program, Research Institute of McGill University Health Centre, Montreal, Quebec, Canada; 10 Department of Cancer Genetics and Genomics, Roswell Park Comprehensive Cancer Center, Buffalo, NY USA; 11 Department of Urology and Urological Science Institute, Yonsei University College of Medicine, Seoul, Republic of Korea; 12 Department of Pathology, Yonsei University College of Medicine, Seoul, Republic of Korea

**Keywords:** Cancer metabolism, Stress signalling

## Abstract

Clear cell renal cell carcinoma (ccRCC) is characterized by profound metabolic dysregulation, with both prolyl hydroxylase domain protein 3 (PHD3) and pyruvate carboxylase (PC) independently implicated in disease progression. Although each influences patient outcomes, a direct mechanistic interplay between these two regulators has remained elusive. Here, we uncover a novel regulatory axis involving PHD3 and PC by identifying an unexpected subcellular behavior of PHD3, namely, its dual localization to the cytosol and the mitochondrial matrix. We show that mitochondrial import of PHD3 is associated with its intracellular clustering, a process modulated by PHD3 hydroxylase activity and oxygen levels. Once in the matrix, PHD3 directly hydroxylates PC, suppressing its enzymatic activity. In ccRCC with elevated PHD3 expression, this modification restricts anaplerotic flux into the tricarboxylic acid cycle, leading to impaired proliferation, reduced metastasis, and enhanced apoptosis. Together, our findings provide a new framework for targeting cancer metabolism by establishing a previously unrecognized mechanistic link between PHD3-mediated oxygen sensing within the tumor microenvironment and the regulation of ccRCC mitochondrial metabolism through the subcellular re-localization of PHD3.

## PHD3 and PC reveal new cancer metabolism pathway

Clear cell renal cell carcinoma (ccRCC) is a formidable cancer, often diagnosed at advanced stages with poor survival rates. This study explores the role of prolyl hydroxylase domain protein 3 (PHD3) in ccRCC, focussing on its interaction with pyruvate carboxylase (PC), a key enzyme in cancer metabolism. The authors used various cell lines and advanced techniques such as confocal microscopy and mass spectrometry to investigate localization and function of PHD3. They discovered that PHD3 re-locates to mitochondria, where it hydroxylates PC, inhibiting its activity and reducing cancer cell growth and invasion. This inhibition limits the anaplerotic flux into the tricarboxylic acid cycle, a crucial metabolic pathway for cancer cells. The study highlights PHD3 as a potential therapeutic target, suggesting that targeting the PHD3–PC axis could offer new treatment strategies for ccRCC by exploiting its metabolic vulnerabilities. This summary was initially drafted using artificial intelligence, then revised and fact-checked by the author.

## Introduction

Clear cell renal cell carcinoma (ccRCC) is a challenging and aggressive cancer, often diagnosed at advanced stages in which the 5-year survival rates drop markedly from more than 90% in localized disease to as low as 12% for metastatic cases^1,2^. A hallmark of ccRCC is its distinct metabolic profile, characterized by impaired oxidative phosphorylation and enhanced glycolysis^3^. To meet their biosynthetic and proliferative demands under these conditions, ccRCC cells increasingly rely on anaplerotic pathways to replenish tricarboxylic acid (TCA) cycle intermediates. A key enzyme supporting this metabolic adaptation is pyruvate carboxylase (PC), a mitochondrial matrix enzyme that converts pyruvate to oxaloacetate. Elevated PC activity has been associated with tumor growth and metastasis^4,5^. PC becomes particularly essential when mitochondrial respiration is compromised, such as in tumors with succinate dehydrogenase deficiency, a common feature in von Hippel–Lindau (VHL)-deficient ccRCC^6,7^.

Alongside PC, prolyl hydroxylase domain protein 3 (PHD3) is another key player implicated in ccRCC progression, with PHD3 expression frequently altered, particularly in tumors carrying VHL mutations^8,9^. PHD3 belongs to a family of oxygen-sensitive enzymes that mediate cellular responses to hypoxia arising within the tumor microenvironment (TME). In ccRCC, the TME is characterized by aberrant vasculature, high metabolic demand, and impaired oxygen diffusion, all of which generate persistent and spatially heterogeneous hypoxic niches. Within these hypoxic microdomains, PHD3 regulates cellular adaptation by hydroxylating proline residues on substrates such as hypoxia-inducible factor-1 (HIF-1)α and HIF-2α^10,11^. Although VHL loss leads to elevated HIF signaling, recent studies suggest that PHD3 suppression may promote tumor progression independently of the canonical VHL–HIF axis, implying an uncharacterized regulatory mechanism^12,13^. Furthermore, evidence that PHD3 contributes only modestly to canonical HIF-1α regulation and displays unique oxygen sensitivity relative to other PHD isoforms further supports the existence of a HIF-1α-independent pathway^10,14^.

Notably, PHD3 has been implicated in alternative cellular pathways, including the control of apoptosis and metabolism^15,16^. Previous proteomic analyses using affinity purification–mass spectrometry (MS) have identified several physical interactors of PHD3, including many mitochondrial proteins. Specifically, a mitochondrial outer membrane-localized acetyl-CoA carboxylase 2 (ACC2), a regulator of fatty acid oxidation, was characterized as a prominent substrate of PHD3^17,18^. PHD3-dependent hydroxylation of ACC2 inhibits its activity and suppresses cancer cell proliferation^19^. Although the outer mitochondrial membrane-localized ACC2 can gain direct access to PHD3, many other potential targets of PHD3 including PC are mitochondrial matrix-resident proteins. This raises key questions about whether these potential interactions can happen despite the compartmental separation between cytosolic PHD3 and mitochondrial matrix-resident proteins and whether these interactions have any biological relevance.

In this study, we address these unresolved questions by identifying the precise mechanism that enables oxygen availability within the TME to directly regulate mitochondrial metabolism in ccRCC. We show that oxygen-sensitive intracellular distribution of PHD3 is associated with its import into the mitochondrial matrix, establishing a physical route through which hypoxic TME signals can access and control intramitochondrial enzymes. Once imported, PHD3 hydroxylates PC and suppresses its enzymatic activity. This oxygen-dependent regulatory mechanism restricts anaplerotic carbon flux into the TCA cycle, thereby limiting the proliferative and metastatic capacity of ccRCC cells. Our findings reveal an unrecognized mitochondrial function of PHD3 and highlight the therapeutic potential of targeting the PHD3–PC axis in kidney cancer.

## Methods

### Cell lines and cell culture

HEK293T (human embryonic kidney), HK-2 (non-cancerous renal epithelial cell line), 786-O and Caki-1 (ccRCC), and Renca (murine renal adenocarcinoma) cell lines were used in this study. HEK293T cells were kindly provided by the Haigis Laboratory (Harvard Medical School), who originally obtained them from the American Type Culture Collection (ATCC). HK-2, 786-O, Caki-1, and Renca cells were generously shared by the Won Sik Ham Laboratory (Yonsei University College of Medicine), which originally acquired them from ATCC. All cells were used within 20 passages, authenticated, and confirmed to be free of mycoplasma contamination before use. HEK293T cells were cultured in Dulbecco’s modified Eagle medium (DMEM; Gibco, 11995073), whereas 786-O, HK-2, and Renca cells were maintained in Roswell Park Memorial Institute 1640 medium (RPMI 1640; Gibco, 11875119). Caki-1 cells were cultured in McCoy’s 5A Medium (Gibco, 11995073). All media contained 4.5 g/l (25 mM) glucose and were supplemented with 10% fetal bovine serum (Gibco, 16000044) and 1% penicillin–streptomycin (Gibco, 15140148). Cells were incubated at 37 °C in a humidified atmosphere containing 5% CO_2_. For drug treatment experiments, cells were seeded into 6-well plates at a density of 5 × 10^5^ cells per well. After 20 h, cells were transfected with the indicated original or mutant plasmids for 24 h. The medium was then replaced with fresh drug-containing medium, and cells were incubated for an additional 24 h.

### Preparation of plasmids, short hairpin RNAs, and transfection

PC, methylcrotonyl-CoA carboxylase subunit 1, propionyl-CoA carboxylase subunit alpha (PCCA), and PCC subunit beta (PCCB) cDNA in pDONR223 vector were obtained from the hORFeome v8.1 Collection^20^ (gift from Roswell Park), and pcDNA3.1-HA-PHD3 was obtained from the Marcia Haigis’s Laboratory at Harvard Medical School. PC cDNA was cloned into pcDNA3.1-ccdb-3XFLAG-V5 vector (gift from Mikko Taipale, Addgene, 87063), pDEST-EGFP-ccdB vector, and pDEST-ccdB-mCherry vector (Chan Young Park’s Laboratory at Ulsan National Institute of Science and Technology) using Gateway LR Clonase II Enzyme Mix (Invitrogen), according to manufacturer’s instructions. Briefly, 10 μl reactions containing 150 ng PC pDONR223, 150 ng pcDNA3.1-ccdb-3XFLAG-V5 vector, and 0.5 μl Clonase in TE buffer (pH 8.0) were incubated at 37 °C for 2 h. Site-specific mutations of PC, PHD3, and stop codon deletion constructs were performed using PCR-based mutagenesis (KOD-Plus Mutagenesis Kit, TOYOBO). PCR primers and plasmids are listed in Supplementary Tables 1 and 2, respectively.

For transient transfection with plasmids to overexpress proteins, cells at ~40% confluency were transfected with plasmids using Lipofectamine (Invitrogen). The transfected cells were allowed to be stabilized for 48 h before being used in experiments. The short hairpin RNAs containing a hairpin loop were synthesized and inserted into pLKO-puro vector. The viral vectors were co-transfected into HEK293T cells with pRSV-Rev, pMD2-VSVG, and pMDLg/pRRE plasmids using polyethyleneimine (Polysciences) to prepare viral particles. On the 3rd day after transfection, lentiviruses were collected from the supernatant of HEK293T cells by using 0.45 μm filters. Cells were infected with viruses (100 μl/ml) with 10 μg/ml polybrene and selected using puromycin (2 μg/ml) to establish stable cell lines. Detailed short hairpin RNA sequences were provided in Supplementary Table 1.

### Knockout cell line generation via CRISPR–Cas9

To generate knockout (KO) cell lines, 23-bp target sequences (5′-N_20_NGG-3′) within the exons of human *PHD3* (GenBank ID: 112399) and *AAVS1* (GenBank ID: 4659) and mouse *Phd3* (GenBank ID: 112407) and *Aavs1* (GenBank ID: 320632) were selected. For each target site, oligonucleotides were designed as forward (5′-CACCG-N_20_-3′) and reverse (5′-AAAC-N_20_-C-3′) sequences, in which N_20_ represents the 20-nt target sequence without the protospacer adjacent motif. The targeted sequences are listed in Supplementary Table 1. Oligos were synthesized as unphosphorylated 25-base polymer/oligonucleotides, annealed by heating at 95 °C for 5 min and gradually by cooling to room temperature. The annealed oligos were cloned into the pSpCas9(BB)-2A-Puro (PX459) V2.0 vector via Golden Gate assembly^21^. Assembly reactions (15 µl total) contained 2 µl of annealed oligos (50 µM), 2 µl of vector (100 ng/µl), 1.5 µl of 10× T4 DNA ligase buffer, 1.5 µl of 10× restriction enzyme buffer, 1 µl of BbsI (NEB), 1 µl of T4 DNA ligase (NEB), and nuclease-free water. The reaction was incubated at 37 °C for 5 h, followed by heat inactivation (50 °C for 5 min and 80 °C for 10 min), and transformed into DH5α-competent cells for plasmid amplification. Cells were seeded and, after 20 h, transfected with the constructed plasmids. About 24 h post-transfection, cells were treated with puromycin (2 µg/ml) for 3 days to select for KO cells.

### Quantitative RT-PCR analysis

Total RNA was isolated from cells using Trizol reagent according to the manufacturer’s instructions (Invitrogen) or with the RNA Clean & Concentrator Kit (Zymo Research). cDNA was synthesized using HiSenScript^TM^ RH(-) RT-PCR PreMix Kit (Lilif). Quantitative real-time PCR was performed with SYBR Green Q-PCR Master Mix (SmartGene) on a Lightcycler 480 instrument (Roche). Data were analyzed using the ΔΔ*C*_t_ calculations, and expression in cell lines was normalized to the reference gene B2M. All primer sequences are provided in Supplementary Table 1.

### Immunoprecipitation and western blotting

Unless otherwise specified, cells were lysed in 1% NP40 buffer containing protease and phosphatase inhibitors. For PC hydroxylation time-course studies, the pan-PHD inhibitor dimethyloxaloylglycine (1 mM, Frontier Scientific) was added to the lysis buffer to prevent further hydroxylation in the lysate. For immunoprecipitations, lysates from cells overexpressing tagged proteins were incubated with EZview anti-HA Affinity Gel (Sigma) or antiFlag Affinity Gel (Sigma). Protein bands were visualized using enhanced chemiluminescence detection reagents (PerkinElmer). Detailed information about the antibodies and reagents used in this study is available in Supplementary Tables 3 and 4, respectively.

### Confocal fluorescence microscopy

To visualize fluorescently tagged proteins, cells expressing fluorescent protein-tagged constructs were cultured on glass coverslips to 40–60% confluency. The cells were then rinsed with phosphate-buffered saline (PBS) and fixed with 3.7% paraformaldehyde in PBS for 15 min at room temperature. Following fixation, cells were permeabilized with 0.5% Triton X-100 in PBS for 5 min and blocked for 30 min at 25 °C, with PBS containing 2% bovine serum albumin (BSA). After three washes in PBS, coverslips were mounted onto slides using ProLong Gold Antifade reagent (Invitrogen, P36941) and imaged using an LSM980 confocal microscope (Carl Zeiss).

### Cell viability assay

Cell viability was assessed using two separate methods. All experiments were performed in at least technical triplicates.

For luminescence-based viability assay, cells were plated in 96-well plates. At the indicated time points, viability was determined using the CellTiter-Glo Luminescent Cell Viability Assay (Promega, G7572) according to the manufacturer’s instructions. Luminescence was measured using a Tecan M200 multiwell plate reader with a 1,000 ms integration time per well.

For fluorescence-based viability experiments, cells were seeded in 96-well plates at a density of 1,000 cells per well and cultured for up to 3 days. To measure viability as a proxy of proliferation, 10 µl of MAX-BLUE reagent (BIOMAX, BCV-R1000) was added to each well and incubated for 1 h at 37 °C. Fluorescence was measured using a BIOTEK Synergy HT plate reader (Ex/Em = 530/590 nm). For data analysis, the fluorescence value from background wells (blank samples treated with 10% NP40 solution to represent 0% viability) was subtracted from all sample values. These adjusted values were then normalized to the fluorescence measurement at the 0-h time point.

### Crude mitochondrial fractionation

Crude mitochondrial fractionation was conducted following a protocol from ref. ^22^. Briefly, cells were lysed on ice using mitochondria isolation buffer (MIB: 1.5 mM Tris–HCl (pH 7.4), 60 mM KCl, 15 mM NaCl, 5 mM MgCl_2_, 1 mM CaCl_2_, 250 mM Sucrose, 100× Phosphatase inhibitor cocktail (GEN DEPOT), and 5 mM EDTA). Lysates were passed through a 25-G needle to disrupt cell membranes and then centrifuged at 1,000×*g* for 5 min at 4 °C to remove the nuclear fraction. The supernatant containing cytosol and mitochondria was further centrifuged at 15,000×*g* for 10 min at 4 °C. The resulting mitochondrial pellet was washed with MIB to remove residual cytosolic contamination. After the final wash, the mitochondrial pellet was lysed with 100 μl RIPA buffer for subsequent protein assays and western blot analysis.

### Proteinase K protection assay

Proteinase K protection assay was conducted following a modified method as described previously^23^. Briefly, cells were seeded in 150 mm plates, cultured overnight, and then transfected with plasmids encoding *EGLN3*, *PC*, or *ACC2*. At 48 h post-transfection, cells were harvested in MIB as described in the crude mitochondrial fractionation method. Each lysate was divided into six equal aliquots and centrifuged at 16,000×*g* for 30 min at 4 °C to obtain crude mitochondrial pellets.

Three mitochondrial pellets underwent the following treatments: (1) resuspension in 500 µl of MIB and incubation on ice for 10 min for untreated mitochondria; (2) resuspension in 500 µl of MIB containing 20 µg/ml of proteinase K and incubation on ice for 10 min for digestion of surface-exposed outer mitochondrial membrane proteins; and (3) incubation with 400 µl of 1% (v/v) Triton X-100 in MIB on ice for 20 min, followed by incubation with 500 µl of MIB containing 20 µg/ml of proteinase K on ice for 10 min for digestion of all mitochondrial proteins. The other three pellets were subjected to osmotic shock by resuspension in 250 µl of a hypotonic solution (5 mM HEPES, 5 mM sucrose, 1 mM EGTA, pH 7.4) for 10 min on ice, followed by addition of an equal volume of a hypertonic solution (750 mM KCl, 80 mM HEPES, 1 mM EGTA, pH 7.4) to re-establish isotonic conditions. The suspension was centrifuged at 3,000×*g* for 15 min at 4 °C to obtain mitoplasts (pellet). These three mitoplast pellets underwent the same treatments as described earlier. Following the last incubation of each treatment, TCA precipitations were collected and solubilized with 400 µl of SDS sample buffer.

### PC activity assay

PC activity was measured using a Pyruvate Carboxylase Activity Assay Kit (Solarbio Life Sciences, BC0730) following the manufacturer’s instructions. Briefly, cells were washed with PBS and scraped into 1 ml of the kit’s extraction buffer. The cell suspension was homogenized on ice and then centrifuged at 1,000×*g* for 10 min at 4 °C to remove insoluble cell debris. The supernatant was transferred to a new tube and centrifuged at 11,000×*g* for 15 min at 4 °C. The pellet was resuspended in 1 ml of extraction buffer and sonicated on ice (20 W output, 12 cycles of 5 s pulses with 10 s intervals).

The assay was prepared by combining 10 μl of the sonicated sample or distilled water as a blank and 190 μl of the working solution (containing malate dehydrogenase, acetyl-CoA, and NADH) in a 96-well plate. The initial absorbance values (AT_1_ or AB_1_) and the absorbance values after 2 min (AT_2_ or AB_2_) were then detected using a BIOTEK Synergy HT microplate reader at 340 nm. PC activity was calculated by converting the change in absorbance ((AT_1_ – AT_2_) – (AB_1_ – AB_2_)) into nmol/min per 10^4^ cells.

### Metabolite profiling and mass spectrometry

Metabolites were isolated in 80% MeOH and analyzed on two distinct methods of hydrophilic interaction liquid chromatography coupled to MS (HILIC-MS). In one method, electrospray ionization was tailored to negative-ion mode, and in the second method to positive-ion mode. For negative-ion mode, analytes were eluted in buffer A (20 mM ammonium acetate, 20 mM ammonium hydroxide) and buffer B (10 mM ammonium hydroxide in 75:25 acetonitrile:methanol). Samples were run on a HILIC silica (3 μm, 2.1 × 150 mm) column (Waters) with a binary flow rate of 0.4 ml/min for 10 min on linear gradient (95% buffer B to 0% buffer B) followed by 2 min (0% buffer B) and ending with a 2 min linear gradient (0% buffer B to 95% buffer B) and holding (95% buffer B) for 13 min. For positive-ion mode, samples were dried down and reconstituted in acetonitrile:methanol:water (20:70:10, v/v/v). The buffers were: buffer A (10 mM ammonium formate, 0.1% formic acid in water) and buffer B (acetonitrile, 0.1% formic acid). Samples were run on a HILIC silica (3 μm, 2.1 × 150 mm) column (Waters) with a binary flow rate of 0.25 ml/min for 10 min on linear gradient (95% buffer B to 40% buffer B) followed by 4.5 min (40% buffer B) and ending with a 2 min linear gradient (40% buffer B to 95% buffer B) and holding (95% buffer B) for 13 min. For both ion modes, a Q Exactive hybrid quadrupole orbitrap mass spectrometer (Thermo Fisher Scientific) with a full-scan analysis over 70–800 *m*/*z* and high resolution (70,000) was used for mass detection. A targeted method developed for 176 compounds (118 on positive and 58 on negative) was used to identify metabolites. A master mix of reference standards for metabolites in the targeted method was run immediately before each set of samples, such that their retention times were associated with peaks in the unknown samples run over that same column. Peaks were integrated in Tracefinder 3.3. Metabolite levels were normalized to cell number in parallel plates or protein concentration in the same amount of tissue powder samples.

### LC-MS/MS-based identification of hydroxylated peptides

Mass spectrometric data were collected using a Q-Exactive Plus mass spectrometer (Thermo Fisher Scientific, Bremen, Germany) coupled with a UktiMate 3000 Autosampler (LC Packings) and an DIONEX liquid chromatography pump (Thermo Fisher Scientific). Peptides were separated on a 75-μm inner diameter microcapillary column packed with 50 cm of RSLC C18 resin (2 μm, 100 Å, PepMAP, Thermo Fisher Scientific). For each analysis, separation was achieved using a 190 min gradient of 3–97% acetonitrile in 0.08% formic acid at a flow rate of 0.3 nl/min. Q-Exactive PLus data were collected using a high-resolution MS2 (hrMS2) method. The scan sequence began with an MS1 spectrum (Orbitrap resolution, 70,000; mass range 400−1500 Th; automatic gain control target, 3 × 106; maximum injection time, 100 ms). MS2 analysis consisted of high-energy collision-induced dissociation with the following settings: resolution, 17,500; automatic gain control target, 1 ×105; isolation window 2 *m*/*z*; normalized collision energy, stepped: 27; and maximum injection time, 100 ms.

### Wound healing and transwell migration assays

The wound healing assay was conducted as described previously^24^, with the following modifications. Briefly, following the transfection with *EGLN3, PC*, and pcDNA 3.1 3XFLAG V5 empty vector for 24 h, cells from each plate were seeded into 6-well plates to reach 95% confluency. A 200 μl pipette tip was used to create a vertical scratch on the culture dish, followed by two to three washes with PBS to remove detached cells. Each well was then observed and photographed under an inverted microscope at 0 h and 8 h, and the scratch characteristics were measured using ImageJ software. The cell migration rate was calculated using the formula: [(width 0 h – width 8 h)/width 0 h] × 100%.

The transwell migration assay was also conducted following a previously described method^24^, with modifications, using 8 μm pore size chambers (Corning, CLS 3422). Cells were serum-starved overnight in FBS-free RPMI 1640 medium. Subsequently, 3 ×10^4^ cells/well were resuspended in 200 μl of serum-free medium and inoculated into the upper chamber, whereas the lower chamber contained 500 μl of RPMI 1640 medium containing 10% FBS. Cells were incubated at 37 °C in a 5% CO_2_ incubator for 24 h for migration assay. Cells in the upper chamber were carefully removed and cells on the surface of the lower chamber were fixed with 4% paraformaldehyde, stained with crystal violet, and washed with Dulbecco’s PBS. Five randomly selected fields of view were examined under a light microscope and the migrating cells were photographed and counted.

### Mitochondrial targeting sequence prediction analysis

Mitochondrial targeting peptide cleavage sites, probability scores, and physicochemical properties of PC and PHD3 were determined using MitoFates (https://mitf.cbrc.pj.aist.go.jp/MitoFates/cgi-bin/top.cgi). A consensus for the mitochondrial targeting sequence (MTS) of mitochondrial proteins, including PC, was generated by WEBLOGO, and mitochondrial protein list was obtained from ref. ^25^ (https://weblogo.berkeley.edu/logo.cgi).

### Molecular dynamics simulations

To investigate the structural stability and dynamics of the PHD–substrate complexes, molecular dynamics (MD) simulations were conducted using the Desmond package. Each system was solvated with the TIP3P water model and enclosed within an orthorhombic periodic boundary box to define simulation boundaries. Counter ions were added to neutralize charges, and the salt concentration was maintained at a physiological level of 0.15 M. The systems underwent energy minimization to ensure optimal starting conformations, followed by a 100-ns MD simulation using the constant pressure and temperature (NPT) ensemble. Pressure was maintained at 1.01 bar using Martyna–Tobias–Klein barostats, and temperature was regulated at 303 K with a Nose–Hoover thermostat. Simulation trajectories were recorded at 20-ps intervals to capture detailed interaction dynamics between PHD and its substrates.

### Binding free energy calculation

Binding free energy was calculated from MD trajectories using the molecular mechanics/generalized Born surface area method implemented in the Prime software. Conformations were sampled every 2 ns over the 100-ns trajectory. The VSGB 2.0 solvent model, which includes empirical corrections for hydrogen bonding and π-stacking interactions, was used to ensure accurate free energy estimates. This approach allowed the assessment of interaction strength, stability, and energetic favorability of the protein–substrate complexes.

### Fluorescence recovery after photobleaching

Fluorescence recovery after photobleaching (FRAP) experiments were performed using a modified protocol based on previously described methods^26^. HEK293T cells were seeded in 6-well plates and transfected with EGFP-tagged PHD3 constructs (wild-type, H196A, and R205K). Six hours after transfection, cells were re-seeded onto glass-bottom dishes (SPL, 33296). Eighteen hours after transfection, cells were stained with 500 nM MitoTracker Orange (Invitrogen, M7510) for 30 min at 37 °C, washed, and replaced with pre-warmed DMEM before imaging.

Imaging was conducted using a Zeiss LSM800 META confocal microscope equipped with Physiology software (Carl Zeiss). A region of interest (ROI) within fluorescent puncta was selected and imaged (prebleach), followed by photobleaching using 488 nm argon laser at 25% power for 20 iterations. Fluorescence recovery was subsequently recorded at 2% laser power at 2s intervals. Recovery kinetics and corresponding graphs were analyzed using Zeiss zen 3.10.

### Quantitative FRAP analysis

For quantitative evaluation of fluorescence recovery, the fluorescence intensity of the bleached ROI was monitored over time. To analyze the recovery kinetics, three key intensity parameters were determined for each cell. The prebleach intensity (*I*_pre_) was calculated as the mean intensity before the photobleaching event. The bleach intensity (*I*_bleach_) was defined as the minimum intensity immediately following the bleach pulse. The plateau intensity (*I*_plateau_) was determined by averaging the fluorescence intensity values during the final steady-state phase of the recovery curve.

The mobile fraction, representing the proportion of molecules capable of dynamic exchange, was calculated using the equation of mobile fraction (%) = (*I*_plateau_−*I*_bleach_)/(*I*_pre_−*I*_bleach_) x 100.

The recovery half-time (*t*_1/2_) represents the time required for the fluorescence intensity to recover to half of its maximum plateau value. This was determined by finding the time point corresponding to the half-maximal intensity, defined as *I*_half_ = *I*_bleach_ + (*I*_plateau_ − *I*_bleach_)/2. For precise *t*_1/2_ estimation, linear interpolation was applied between the adjacent time points bounding *I*_half_. For catalytically inactive variants that exhibited solid-like behavior with near-complete loss of mobility (immobile fraction > 85%), the *t*_1/2_ was designated as not determined owing to the absence of a meaningful recovery phase. All quantitative data are presented as mean ± standard deviation (SD) derived from independent biological replicates (*n* = 3 cells per condition).

### Colocalization analysis

Colocalization analysis was performed using Fiji with the Coloc2 plugin. ROIs were manually defined on a per-cell basis. Manders’ colocalization coefficients were calculated using the automatic thresholding method implemented in Coloc2 (Costes’ automatic threshold), yielding thresholded Manders’ coefficients (tM1 and tM2).

The mitochondrial compartment was defined based on the thresholded TMRM channel, and colocalization between PHD3–GFP and TMRM signals was quantified within this mitochondrial mask. For statistical analysis and graphical presentation, tM2 values, which represent the fraction of the TMRM signal overlapping with PHD3–GFP after thresholding, were used.

### Seahorse XF analysis

786-O cells were transfected with a PHD3 expression construct. Twenty-four hours post-transfection, cells were seeded into XF 96-well microplates at a density of 7000 cells per well. The following day, cells were treated with ZY-444 (0.5 μM or 2.5 μM) or CPI-613 (10 μM). After 24 h of treatment, the culture medium was replaced with Seahorse XF RPMI assay medium supplemented with 10 mM glucose, 1 mM pyruvate, and 2 mM glutamine. Cells were incubated at 37 °C in a non-CO_2_ incubator before analysis. Basal oxygen consumption rate (OCR) was measured using a Seahorse XF analyzer. OCR values were normalized to optical density measured for each well.

### Human tissue specimens and histological analyses

Formalin-fixed, paraffin-embedded (FFPE) tissue samples of ccRCC used in this study were obtained from patients who underwent nephrectomy at Yonsei University Severance Hospital between October 2024 and August 2025. All surgical specimens were classified according to the TNM classification and the ISUP grading system. Twelve cases of T2a-4 ccRCC, grades 2–4, were randomly selected for the study. FFPE tissue blocks were sectioned at 4-μm thickness and stained with hematoxylin–eosin, and all slides were independently reviewed by a board-certified genitourinary pathologist (Yonsei University Severance Hospital) to confirm histopathologic features. The human ccRCC tissue specimens used in this study were collected under a prospective clinical specimen collection protocol approved by the Institutional Review Board (IRB) of Yonsei University College of Medicine (IRB approval number: 4-2019-1039). All procedures involving human subjects were conducted in accordance with institutional guidelines and the Declaration of Helsinki. Informed consent was obtained from all patients.

### Immunohistochemistry and immunofluorescence staining

FFPE sections (4 μm) were deparaffinized and subjected to antigen retrieval using 10 mM sodium citrate buffer (pH 6.0). For immunofluorescence (IF) staining, sections were permeabilized and blocked with 5% BSA and 0.3% Triton X-100 for 30 min and then incubated overnight at 4 °C with primary antibodies against PHD3 (Novus, NB100-139) and ATPB (Abcam, ab14730). The samples were incubated with fluorochrome-conjugated secondary antibodies in the dark for 1 h at room temperature and mounted with antifade mounting medium containing 4′,6-diamidino-2-phenylindole. Images were obtained using a confocal laser scanning microscope (LSM780; Carl Zeiss) and analyzed with Zen software (Carl Zeiss).

For immunohistochemistry (IHC), sections were incubated with 3% H_2_O_2_ for 10 min to block endogenous peroxidase and treated with 5% BSA and 0.3% Triton X-100 for 30 min. Then, the samples were incubated overnight at 4 °C with primary antibodies against E-cadherin (Abcam, ab3195) and HIF-1α (Cell signaling, 36169). The sections were then incubated with biotin-conjugated secondary antibodies and visualized using a streptavidin-biotin staining system (Abcam, ab64264). Images were obtained using a digital slide scanner ZEISS Axioscan 7 at Korean Institute of Oriental Medicine.

### RNA-seq dataset analysis

Bulk RNA-seq data and prognostic data for Kidney Renal Clear Cell Carcinoma (KIRC) were obtained from The Cancer Genome Atlas (TCGA-KIRC, https://portal.gdc.cancer.gov/projects/TCGA-KIRC) for downstream bioinformatics analysis. Clinical information was available for 537 patients, and gene expression profiles were obtained from 614 patient samples used in this study. Transcriptome-wide differential expression was assessed using the limma-trend method on log_2_(CPM) data. For clinical subgroup comparison (for example, M0 versus M1), differential gene expression was performed using DESeq2 on raw count matrices, allowing for accurate modeling of dispersion across biological replicates.

### Survival analysis

Survival analysis was performed using the Kaplan–Meier method to estimate overall survival probabilities. The high-expression group was defined as the top 30% of patients ranked by expression level, whereas the remaining 70% were assigned to the low-expression group. The log-rank test was used to evaluate statistical significance between the survival curves. The analysis and visualization were conducted using the survival (version 3.8-3) and survminer (version 0.5.0).

### TCGA and DepMap data analysis

PHD3 (*EGLN3*) gene expression and overall survival data for patients with ccRCC were obtained from TCGA-KIRC via The Human Protein Atlas (https://www.proteinatlas.org/). For correlation analysis, patients within the top 10% of PC expression were selected. Pearson correlation coefficients and simple linear regression analyses were performed using GraphPad Prism (version 10.2.3) to assess the association between PHD3 expression and overall survival (days). Default settings were used, and *P*-values were calculated using two-tailed *t* tests.

PHD3 gene expression across a wide range of cancer cell lines was retrieved from the Expression Public 24Q4 dataset of the Cancer Dependency Map (DepMap) portal (https://depmap.org/portal/). Transcripts per million (TPM)-normalized expression values were used, and a log2(TPM + 1) transformation was applied for visualization and comparative analysis. Kidney cancer cell lines were categorized into high or low PHD3 expression groups based on the mean expression level observed in normal kidney epithelial cell lines.

### Differential gene expression analysis

Differential gene expression analysis was performed using the limma-trend method (limma package version 3.58.1). First, it calculates CPM on the trimmed mean of *m*-values (TMM)-normalized data using edgeR. Unsupervised hierarchical clustering was performed based on Euclidean distance and the average linkage method using the R package gplots (version 3.1.3.1). Then, it fits a linear model to the log-transformed CPM data using the design matrix. Empirical Bayes moderation was applied to the linear model coefficients. After that, we conducted differentially expressed analysis using indicated genes (*EGLN1-3*, *PCCA*, *PCCB*, *PC*, *MCCC1*, *ACACA*, and *ACACB*) with *P*-value cut-off of 0.01 and log_2_ fold change cut-off of 1.0, which was applied to filter differentially expressed genes at R package EnhancedVolcano (version 1.20.0).

### Single-cell RNA-seq dataset analysis

Unnormalized count data were obtained from Wellcome Sanger Institute (https://www.sanger.ac.uk/collaboration/microenvironment-of-kidney-cancer/) and were scaled using a log transformation. To mitigate bias from unequal cell counts across different broad cell types, we performed a down-sampling procedure. Cells were grouped by the broad_type annotation, and for each group, up to 15,000 cells were randomly selected. If a group contained fewer than 15,000 cells, all cells were retained.

### Differential transcriptome analysis of *EGLN3*-positive and *EGLN3*-negative cells

Among 11,915 RCC cells from single-cell RNA-seq dataset, those with a mean of zero for the biotin carboxylase (BC) domain protein transcriptomic signature were excluded. From the remaining 2417 cells, we classified cells as *EGLN3*-positive if they exhibited non-zero expression of *EGLN3*. Cells with no detectable *EGLN3* transcripts were designated as *EGLN3*-negative. On the basis of this classification, pathway enrichment analysis, mean expression calculation, and the proportion of cells expressing genes related to oxygen sensing and cell migration were evaluated.

### Statistical analysis

Details regarding the specific statistical tests, definition of center, and number of replicates (*n*) can be found for each experiment in the figure legends. Unpaired two-tailed Student’s *t* tests were used for comparison of individual experiments, and statistical significances were considered when *P*-values were less than 0.05. GraphPad Prism and Microsoft Excel were used for all quantifications and statistical analyses. Statistical significance was denoted as follows: *P* < 0.05(*). Detailed software and tool information are provided in Supplementary Table 5.

## Results

### PHD3 expression correlates with suppressed metastatic programs in ccRCC

**Fig. 1:**
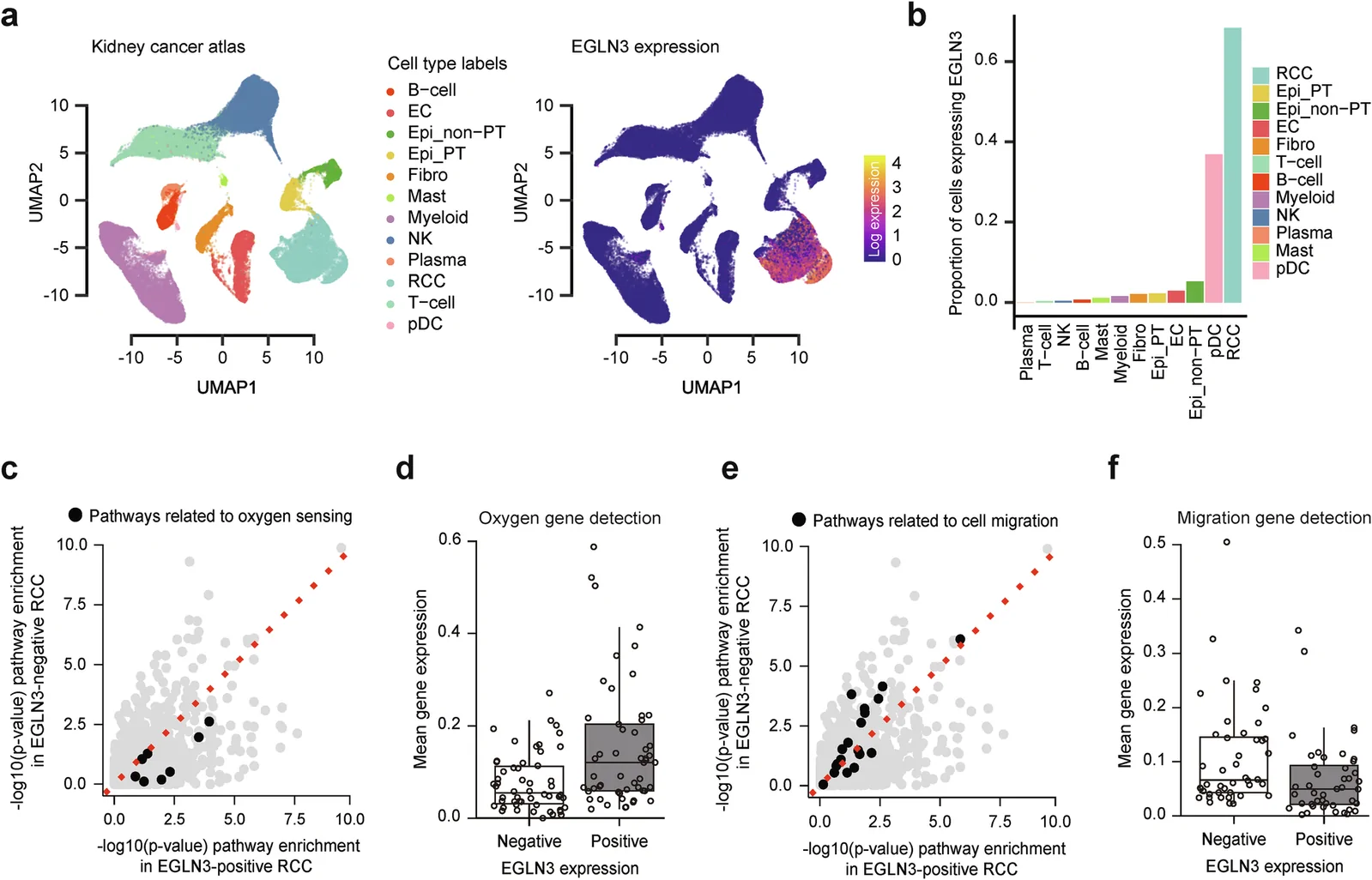
PHD3 expression is associated with suppression of metastatic programs in ccRCC. **a** UMAP representation of single-cell transcriptomes from the kidney cancer atlas. Cells are colored by cell type (left) or *EGLN3* expression level (right). **b** Bar plot showing the proportion of cells expressing *EGLN3* across different cell types. **c** Scatter plot of pathway enrichment analysis, highlighting oxygen-sensing-related pathways in black. **d** Box plot shows the mean expression levels of selected oxygen-sensing genes in *EGLN3*-positive and *EGLN3*-negative cells. **e** Scatter plot of pathway enrichment analysis, with cell migration and epithelial–mesenchymal transition (EMT)-related pathways marked in black. **f** Box plot compares the expression of representative genes involved in migration and EMT between *EGLN3*-positive and *EGLN3*-negative cells. ccRCC, clear cell renal cell carcinoma; PHD3, prolyl hydroxylase domain protein 3; RCC, renal cell carcinoma; UMAP, uniform manifold approximation and projection.

To precisely define the clinical and functional relevance of PHD3 in ccRCC, we first conducted a comprehensive analysis of its expression patterns in patient tumors and their correlation with disease characteristics. To this end, we first analyzed single-cell transcriptomic data from a comprehensive ccRCC atlas to confirm the expression pattern of *EGLN3*, the gene encoding PHD3. *EGLN3* was indeed selectively expressed in renal cancer epithelial cells compared with adjacent normal tissues (Fig. 1a). Among kidney cell types, *EGLN3* expression was predominantly restricted to RCC tumor cells, with 64% of tumor or tumor-adjacent epithelial cells expressing *EGLN3*, in contrast to only 4% in normal epithelial cells (Fig. 1b).

To explore the biological consequences of *EGLN3* expression in RCC cells, we stratified tumor cells into *EGLN3*-positive and *EGLN3*-negative populations and performed differential pathway enrichment analysis. *EGLN3*-positive RCC cells showed significant enrichment of oxygen-sensing and hypoxia-associated pathways, whereas *EGLN3*-negative cells were enriched for gene programs associated with cell migration and metastasis (Fig. 1c–f). At the gene level, *EGLN3*-positive cells expressed higher levels of oxygen-related genes such as *ERO1A, AQP1, AKAP12*, and *CX3CL1* (Fig. 1d and Supplementary Fig. 1a) and lower levels of migratory and epithelial–mesenchymal transition (EMT)-related markers, including *FLNA, CD44, STAT1*, and *PYCR1* (Fig. 1f and Supplementary Fig. 1b). These data suggest that *EGLN3* expression is inversely correlated with metastatic gene signatures and may restrain malignant progression.

Bulk transcriptomic data from TCGA and DepMap showed a similar trend, with PHD3 expression being markedly higher in ccRCC tumor tissue compared with adjacent normal kidney samples (Supplementary Fig. 1c) and significantly upregulated in kidney tumors relative to most other cancer types (Supplementary Fig. 1d). ccRCC cell lines also exhibited elevated PHD3 mRNA and protein levels compared with non-cancerous HEK293T cells (Supplementary Fig. 1e–g).

To evaluate the clinical relevance of PHD3, we stratified patients with ccRCC in the TCGA-KIRC cohort into PHD3-high and PHD3-low groups based on mRNA expression. Kaplan–Meier survival analysis revealed that high PHD3 expression was significantly associated with improved overall survival (Supplementary Fig. 1h). Moreover, PHD3 expression was significantly reduced in metastatic lesions compared with primary tumors within the TCGA dataset (Supplementary Fig. 1i), further supporting its role in suppressing tumor progression.

### PHD3 physically interacts with mitochondrial matrix BC-domain proteins and exhibits inverse expression correlation

**Fig. 2:**
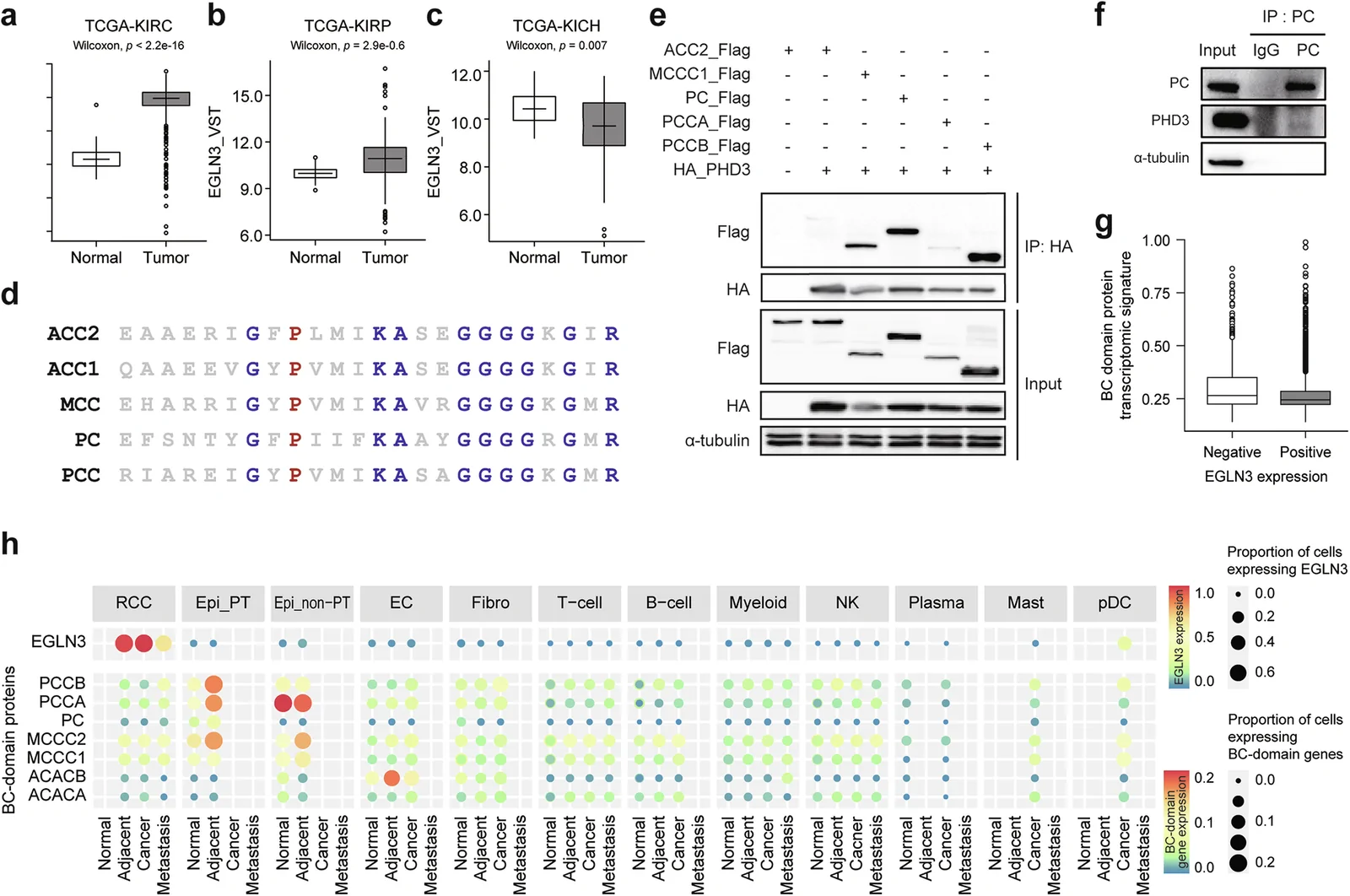
PHD3 binds BC-domain mitochondrial enzymes with inversely correlated expression. **a**–**c** *EGLN3* expression across renal carcinoma subtypes in The Cancer Genome Atlas (TCGA) datasets. Box plots show variance-stabilized counts in normal kidney and tumor samples from KIRC (clear cell renal cell carcinoma (ccRCC)) (part **a**), KIRP (papillary RCC) (part **b**), and KICH (chromophobe RCC) (part **c**). *EGLN3* expression is markedly elevated in ccRCC relative to normal kidney, with modest changes in KIRP and reduced expression in KICH. **d** A consensus sequence alignment of biotin carboxylase (BC) domain-containing proteins (ACC, MCC, PC, and PCC) highlights conserved regions associated with PHD3 binding. **e** HEK293T cell lysates expressing HA-tagged constructs were subjected to immunoprecipitation (IP) using an anti-HA antibody, followed by immunoblotting with the indicated antibodies to detect protein–protein interactions. **f** Endogenous interaction between PC and PHD3 was confirmed by IP in 786-O cells, followed by western blot analysis. **g** Box plot comparing BC-domain gene expression signatures between *EGLN3*-negative (white) and *EGLN3*-positive (gray) renal cell carcinoma (RCC) samples. Statistical significance was determined using a two-tailed Student’s *t* test. **h** Dot plot showing cell-type-specific expression profiles of *EGLN3* and BC-domain-containing genes. Cells were categorized into four microenvironmental regions: normal, cancer adjacent, tumor core, and metastatic sites. Dot color represents average gene expression, and dot size indicates the proportion of cells expressing each gene within the group. ACC, acetyl-CoA carboxylase; MCC, methylcrotonyl-CoA carboxylase; PC, pyruvate carboxylase; PCC, propionyl-CoA carboxylase; PHD3, prolyl hydroxylase domain protein 3.

Following our confirmation of the clinical relevance of PHD3 expression in patient survival in ccRCC, we further investigated the underlying molecular mechanisms responsible for this association. We next assessed whether this axis is selectively enriched in ccRCC. TCGA analysis showed that *EGLN3* expression is markedly elevated in KIRC relative to normal kidney, with only modest changes in papillary RCC (KIRP) and reduced expression in chromophobe RCC (KICH) (Fig. 2a–c). Principal component analysis further demonstrated clear separation among renal carcinoma subtypes, indicating that ccRCC represents a distinct molecular context (Supplementary Fig. 2a). Given that PHD3 functions by hydroxylating specific substrates, we sought to identify the direct targets through which it mediates its effects. To achieve this, we focussed on BC-domain-containing proteins (Fig. 2d). This decision was primarily driven by the fact that the well-characterized PHD3 substrate ACC2 interacts with PHD3 via this domain, and additionally, other BC-domain-containing proteins were identified as putative interacting partners in previous affinity purification–MS screens. These include mitochondrial matrix-localized PC, methylcrotonyl-CoA carboxylase (MCC), PCC, among others.

To this end, we tested whether PHD3 physically interacts with and modifies these candidate enzymes. Co-immunoprecipitation experiments using Flag-tagged BC-domain proteins revealed strong interactions between PHD3 and multiple mitochondrial enzymes, including PC, MCCC1, and PCCB (Fig. 2e). Importantly, we also detected an endogenous interaction between PHD3 and PC, supporting the physiological relevance of this binding (Fig. 2f).

Moreover, the analysis of TCGA data revealed an inverse expression pattern between PHD3 and BC-domain carboxylases in ccRCC tumors, suggesting regulation at the post-transcriptional level as well. Although PHD3 was overexpressed, genes encoding *PC*, *PCCB*, *MCCC1*, and related enzymes were significantly downregulated (Supplementary Fig. 2b, c). This expression pattern was independent of tumor stage or metastatic status (Supplementary Fig. 2d, e).

To validate these trends at single-cell resolution, we stratified tumor epithelial cells based on *EGLN3* expression and computed a BC-domain gene signature score. *EGLN3*-positive cells exhibited significantly lower BC-domain signature expression, supporting a negative association between PHD3 and mitochondrial carboxylase levels (Fig. 2g). Expression of individual carboxylases, including *PC*, *PCCA*, *PCCB*, and *MCCC1*, was also reduced in cancer cells compared with normal epithelial or stromal cells within the TME (Fig. 2h).

Together, these findings suggest a previously unrecognized set of mitochondrial matrix proteins as substrates for PHD3 and highlight their potential importance in ccRCC progression.

### PHD3 localizes to the mitochondrial matrix

**Fig. 3:**
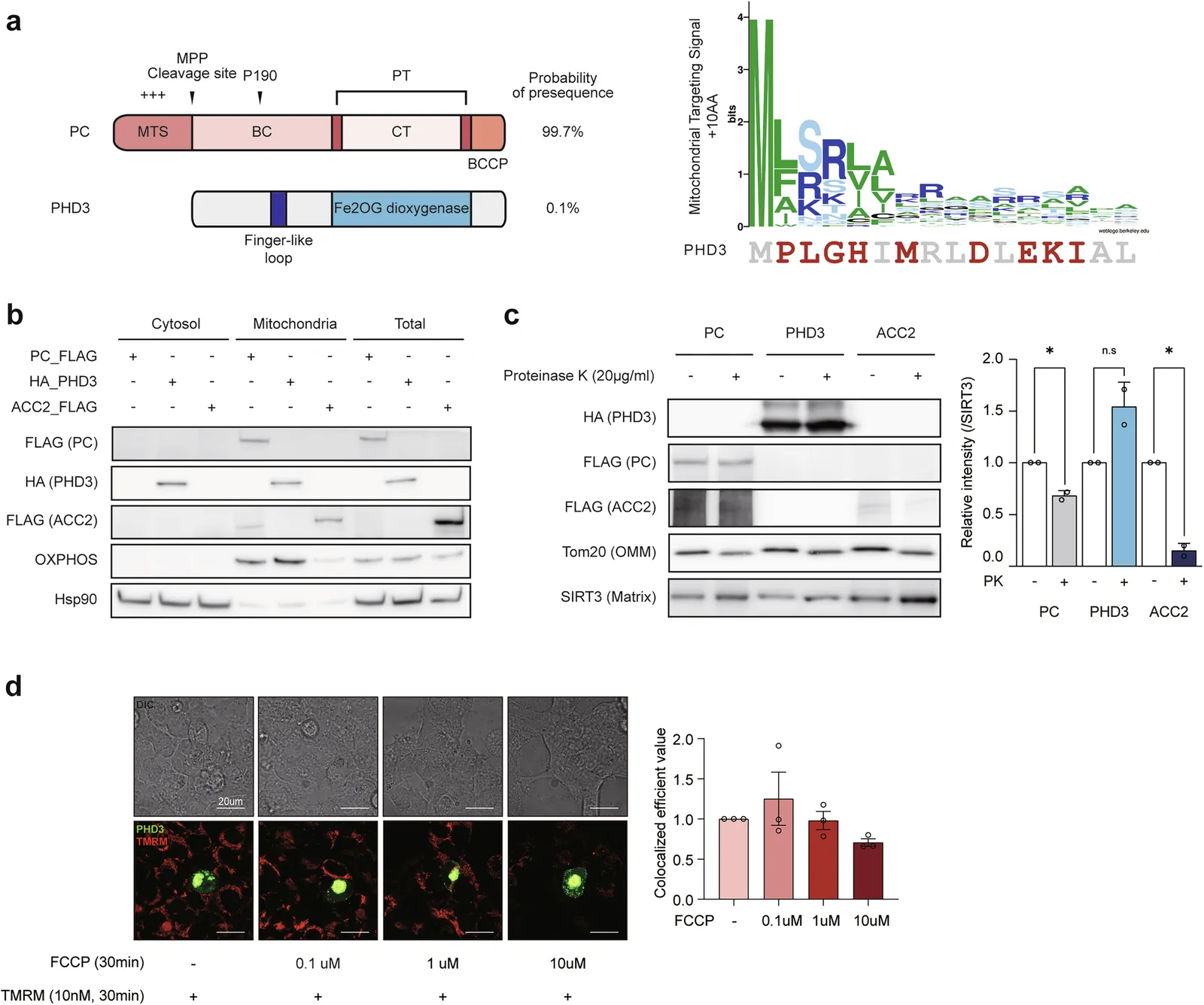
PHD3 localizes to the mitochondrial matrix via a shared targeting motif with PC. **a** Schematic diagrams of pyruvate carboxylase (PC) and prolyl hydroxylase domain protein 3 (PHD3) show protein domains and known post-translational modification sites (left). Mitochondrial targeting sequences (MTS) were predicted using MitoFate (right). The consensus motif for mitochondrial targeting was derived from recent proteomic studies and is shared by both PC and PHD3. **b** Crude mitochondrial fractions were isolated from HEK293T cells, and the subcellular localization of PHD3, PC, and ACC2 was analyzed by western blotting. Oxidative phosphorylation (OXPHOS) and Hsp90 served as markers for mitochondria and cytosol, respectively. **c** A protease protection assay (left) was performed to determine the submitochondrial localization of PHD3, PC, and ACC2, followed by western blot analysis. Quantification of western blot signals from independent experiments (right). **d** FCCP disrupts mitochondrial membrane potential and reduces PHD3 mitochondrial localization. HEK293T cells expressing PHD3-GFP (green) were treated with increasing concentrations of FCCP and stained with TMRM (red). Quantification shows a dose-dependent decrease in TMRM–PHD3 colocalization. Scale bar, 20 μm. The accompanying graph shows dose-dependent changes in TMRM–PHD3 colocalization signals. BC, biotin carboxylase domain; CT, carboxyltransferase domain; MPP, mitochondrial processing peptidase; PCCP, propionyl-CoA carboxylase polypeptide; BC, biotin carboxylase.

Although the mitochondrial matrix enzymes seem to be bona fide substrates of PHD3, such interactions could be a post-lysis artifact when observed in detergent disrupted conditions, as this removes the physical separation between PHD3 and mitochondrial matrix enzymes. Moreover, PHD3 lacks a canonical MTS generally required for the mitochondrial protein import^25^ (Fig. 3a). To address this discrepancy in observations, we first analyzed the subcellular localization of PHD3 by performing high-resolution confocal microscopy in HEK293T cells co-expressing mCherry-tagged PHD3 and EGFP-tagged PC. Notably, PHD3 was detected in both the cytosol and mitochondria, partially colocalizing with mitochondrial marker PC puncta (Supplementary Fig. 3a). Biochemical fractionation also confirmed this observation, with PHD3 detected in both cytosolic and mitochondrial compartments, whereas PC and ACC2 were confined to the mitochondrial fraction only (Fig. 3b and Supplementary Fig. 3b).

To determine whether PHD3 is peripherally associated with mitochondria or imported into the matrix, we performed a proteinase K protection assay (Supplementary Fig. 3c). In intact mitochondria, outer membrane proteins such as ACC2 and VDAC were digested by proteinase K, whereas matrix-localized proteins such as PC, SIRT3, and OXPHOS components were protected. PHD3 was similarly resistant to digestion, indicating localization within the mitochondrial interior (Fig. 3c and Supplementary Fig. 3d). Fraction-specific degradation under various permeabilization conditions confirmed that PHD3 behaves like a matrix protein in terms of protease accessibility. To further examine whether this localization depends on classical MTS-driven, membrane potential-dependent import, we treated PHD3-GFP-expressing cells with FCCP to dissipate Δ*ψ*_m_. Across both dose-response and time-course conditions, loss of TMRM fluorescence confirmed complete depolarization, yet PHD3 mitochondrial localization remained unchanged (Fig. 3d and Supplementary Fig. 3e).

Taken together, these results demonstrate that PHD3 localizes to both the cytosol and the mitochondrial matrix. This localization allows PHD3 to engage directly with matrix-resident metabolic enzymes.

### Oxygen-dependent intracellular distribution of PHD3 is associated with its dual localization to the cytosol and mitochondria

**Fig. 4:**
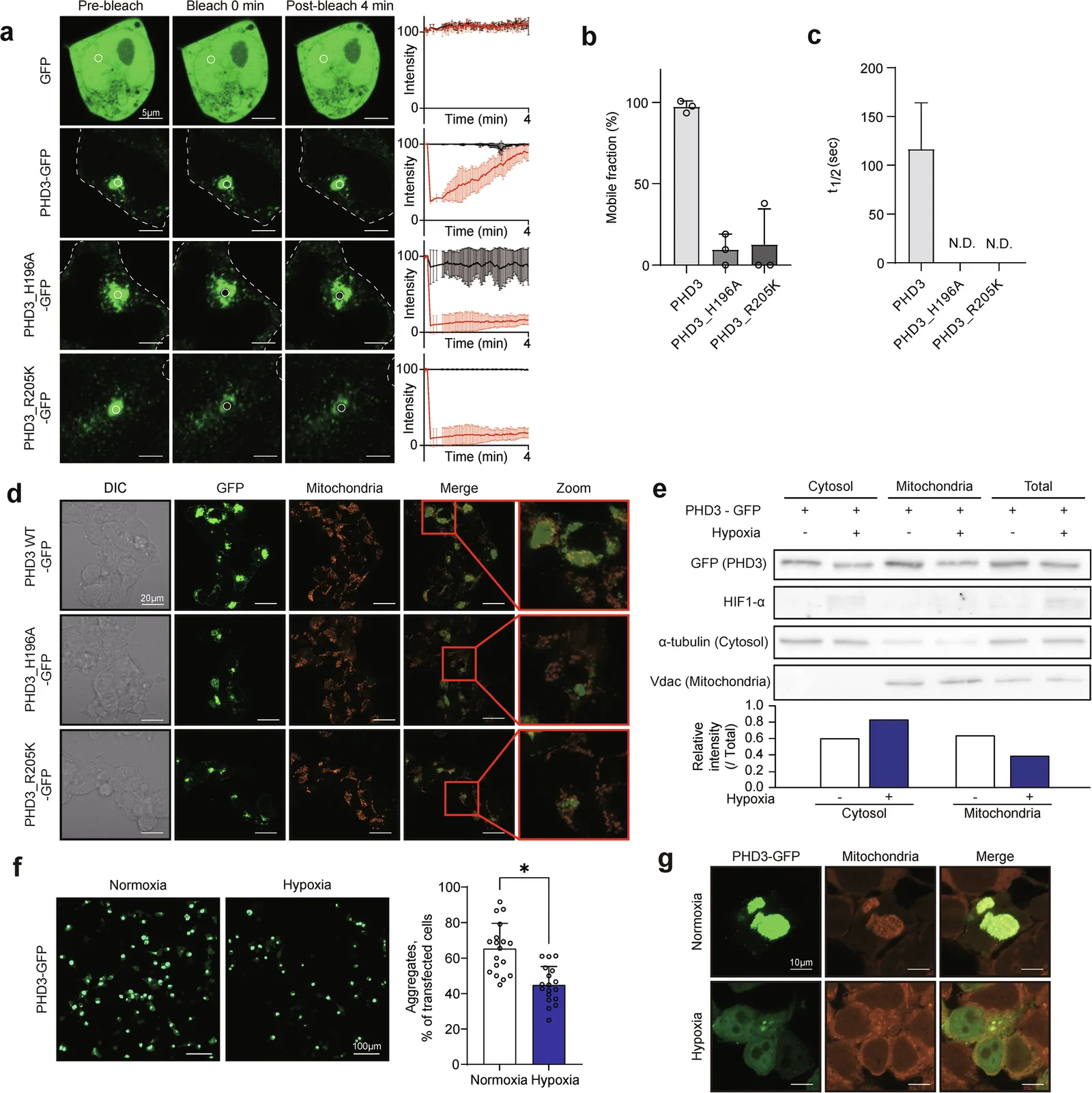
Dynamic properties of oxygen-dependent mitochondrial localization of PHD3. **a** Fluorescence recovery after photobleaching (FRAP) was performed using GFP, prolyl hydroxylase domain protein 3 (PHD3)-GFP, and enzymatically inactive mutants, PHD3 H196A-GFP and PHD3 R205K-GFP (green). Photobleached regions are indicated by white circles. Rapid fluorescence recovery of PHD3-GFP within the bleached aggregates suggests dynamic exchange. Representative FRAP graphs of bleached and unbleached regions are shown. Scale bar, 5 µm. **b** Quantification of the mobile fraction of wild-type (WT) PHD3-GFP and its catalytically inactive variants (H196A and R205K) derived from the FRAP analysis. The data represent the percentage of the mobile pool capable of fluorescence recovery. Data are presented as mean ± SD (*n* = 3 per group). **c** Quantification of the recovery half-time (*t*_1/2_) for WT PHD3-GFP. The *t*_1/2_ represents the time required for the fluorescence intensity to reach half of its maximum recovered plateau. Note: the recovery half-time for the H196A and R205K variants was not determined (ND), as these variants form static structures with a near-complete lack of fluorescence recovery, rendering the calculation of *t*_1/2_ physically uninterpretable. **d** Confocal microscopy shows colocalization of PHD3-GFP and its mutants with mitochondria in HEK293T cells. MitoTracker was used as a mitochondrial marker. Scale bar, 20 µm. **e** Western blot analysis (top) of crude mitochondrial fractions from HEK293T cells cultured under normoxic or hypoxic conditions demonstrates oxygen-dependent mitochondrial localization of PHD3. Quantification (bottom) of western blot signals from a representative experiment as shown in the top. **f** Confocal images (left) of HEK293T cells expressing PHD3-GFP under normoxic and hypoxic conditions, showing decreased aggregation of PHD3 under hypoxia. Quantification of aggregates from a representative experiment is shown in right. Scale bar, 100 µm. **g** Confocal images show mitochondrial colocalization of PHD3-GFP under normoxia and hypoxia. MitoTracker was used to label mitochondria (GFP: excitation/emission 395/475 nm, green; MitoTracker: 552/575 nm, orange). Scale bar, 10 µm. HIF, hypoxia-inducible factor.

We next investigated how PHD3 translocates into the mitochondrial matrix, given its lack of a conventional targeting sequence. To this end, we analyzed GFP-tagged PHD1, PHD2, and PHD3 expressing HEK293T cells. Interestingly, only PHD3 formed discrete cytoplasmic puncta, whereas PHD1 and PHD2 remained diffusely distributed (Supplementary Fig. 4a, b). Quantitative FRAP analysis revealed the diffusive properties of these puncta, as GFP–PHD3 foci showed rapid recovery of fluorescence post-bleach (Fig. 4a). This was surprising as the structural comparison of PHD1, PHD2, and PHD3 revealed that although PHD1 and PHD2 possess substantial N-terminal intrinsically disordered regions (IDRs), which typically mediate condensate formation, PHD3 does not (Supplementary Fig. 4c–e). However, PHD3 forms puncta whereas PHD1 and PHD2 do not. This suggests that PHD3 employs an alternative, IDR-independent mechanism associated with puncta formation, indicating that this behavior may represent a unique feature not shared by other PHD members. Next, we examined whether PHD3 condensation is linked to its enzymatic activity or oxygen levels. Hydroxylase-dead mutants (H196A and R205K), which mimic the hypoxic-inactive state, formed static aggregates with minimal fluorescence recovery after bleaching (Fig. 4a). Furthermore, wild-type PHD3 exhibited highly dynamic exchange properties, characterized by a robust fluorescence recovery with a mean mobile fraction of 97.4% and a recovery half-time (*t*_1/2_) of ~116.5 s (Fig. 4b). In stark contrast, the catalytically inactive H196A and R205K variants displayed a profound loss of mobility (mean mobile fractions of 9.5% and 12.6%, respectively) (Fig. 4c). This near-complete lack of recovery confirms that these variants form functionally static, solid-like structures rather than dynamic assemblies.

These mutants also exhibited markedly reduced mitochondrial localization (Fig. 4d), suggesting that its dynamic state is associated with efficient intracellular distribution and mitochondrial access. Furthermore, biochemical fractionation of cells under normoxic and hypoxic conditions showed significant reduction in mitochondrial levels of PHD3 under hypoxia (Fig. 4e), a trend recapitulated by CoCl_2_ treatment (Supplementary Fig. 4f). Confocal imaging revealed decreased number of cytoplasmic PHD3 puncta under hypoxia (Fig. 4f), coinciding with diminished mitochondrial localization (Fig. 4g). Consistently, analysis of endogenous PHD3 in 786-O cells confirmed a detectable mitochondrial pool that is modestly reduced under hypoxia (Supplementary Fig. 4g), supporting oxygen-dependent regulation at physiological expression levels. This regulation was not observed in non-malignant HK-2 cells (Supplementary Fig. 4h), indicating that it is selectively active in ccRCC. Quantitative analysis of patient-derived ccRCC tissues further showed no differences in mitochondrial area or circularity between PHD3-positive and PHD3-negative mitochondria (Supplementary Fig. 4i,j), excluding structural abnormalities or mitochondrial toxicity.

Together, these findings suggest oxygen-dependent clustering as a key regulatory mechanism that governs the subcellular re-localization of PHD3. By enabling this intracellular distribution under normoxic conditions, it provides a direct molecular link between oxygen sensing and the functional engagement of mitochondrial substrates.

### Mitochondrial PHD3 inhibits PC activity through proline 190 hydroxylation

**Fig. 5:**
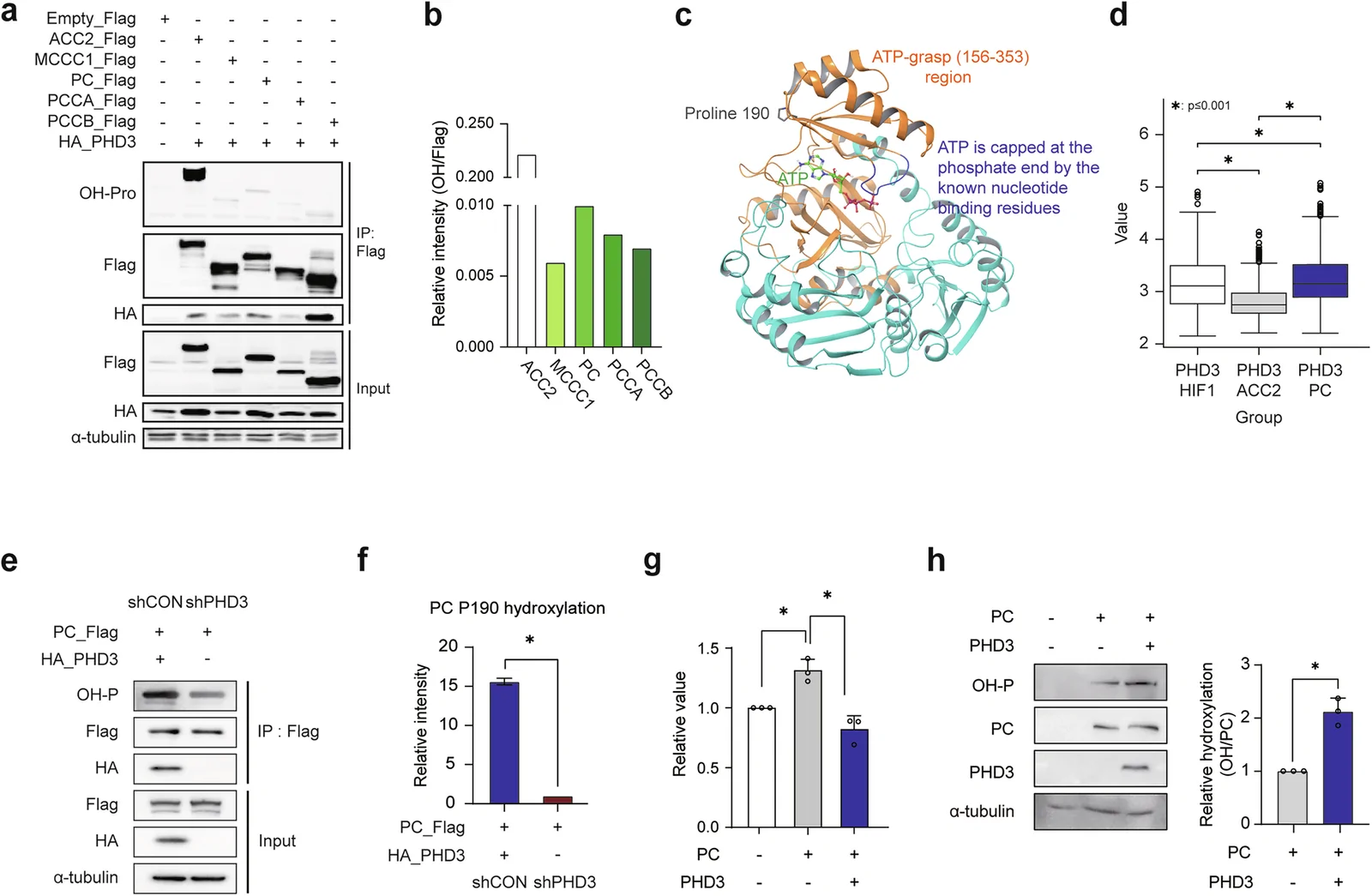
PHD3 directly hydroxylates PC at Pro190 and suppresses its enzymatic activity. **a** HEK293T cell lysates were subjected to immunoprecipitation using an anti-Flag antibody, followed by immunoblotting with the indicated antibodies to assess substrate-specific interactions and hydroxylation using a pan-hydroxyproline antibody. A representative blot from three independent experiments is shown. **b** Quantification of western blot signals from independent experiments as shown in part **a**. **c** Structural model of PC (PDB ID: 7WTB) showing the ATP-grasp domain (residues 156–353) in orange and the remaining domains in blue. ATP (green) and Pro190 (gray) are visualized in the active site. Pro190 is solvent-exposed. The structure is shown with a transparent surface view. ATP is coordinated at the phosphate end by conserved nucleotide-binding residues (blue). **d** Comparative analysis of substrate structures, based on the distance between the α-ketoglutarate cofactor and proline residues, reveals differences in catalytic efficiency. The plot highlights how substrate accessibility and structural flexibility influence PHD3 activity. **e** PC hydroxylation was assessed in HEK293T cells overexpressing or depleted of PHD3. PC was immunoprecipitated and probed by western blot using a hydroxyl-proline-specific antibody. **f** Mass spectrometry analysis identified hydroxylated proline residues in PC from HEK293T cells. Representative spectra show hydroxylation at Pro190 in control cells, which was reduced upon PHD3 knockdown. **g** PC activity assay in HEK293T cells transfected with pcDNA3.1 3XFLAG-V5 (vector control), PC alone, or PC co-expressed with PHD3. Cells were incubated in serum-free medium for 3 h post-transfection. **h** Immunoblot analysis assessing PHD3-dependent hydroxylation of PC under co-expression conditions (left), and quantification of western blot signals from independent experiments (right). ACC2, acetyl-CoA carboxylase 2; HIF, hypoxia-inducible factor; IP, immunoprecipitation; PC, pyruvate carboxylase; PCCA, propionyl-CoA carboxylase subunit alpha; PCCB, propionyl-CoA carboxylase subunit beta; PHD3, prolyl hydroxylase domain protein 3.

To determine the functional consequence of mitochondrial translocation of PHD3, we tested whether the mitochondrial BC-domain-containing enzymes can be hydroxylated by probing for hydroxyproline. ACC2 exhibited the strongest hydroxylation signal as expected, followed by PC and PCCA, suggesting substrate-selective hydroxylation efficiency (Fig. 5a, b). The detection of hydroxyproline in mitochondrial matrix-resident proteins such as PC and PCC supports their identification as bona fide PHD3 substrates, highlighting the physiological relevance of PHD3 and establishing it as a direct target of post-translational regulation.

Upon confirming mitochondrial matrix enzymes as novel substrates of PHD3, we focussed on PC, a key anaplerotic enzyme that converts pyruvate to oxaloacetate to support TCA cycle flux under metabolic stress. PC exhibited the highest hydroxylation efficiency among the mitochondrial substrates. Moreover, PC becomes essential for sustaining mitochondrial metabolism, particularly under glutamine-limited conditions in ccRCC, in which succinate dehydrogenase is frequently impaired^4,7,27^.

To further verify and understand the structural basis of this interaction, we modeled the PHD3–PC complex in silico. The analysis confirmed the interaction of these two proteins and suggested proline 190 (P190) as the primary hydroxylation site. P190 resides at the entrance of the ATP-grasp domain in PC, a position analogous to hydroxylated sites in ACC2, suggesting a conserved mechanism of substrate engagement (Fig. 5c). Furthermore, during MD simulation^28^, PHD3 maintained catalytically competent distances to P190, supporting its role in selective substrate recognition (Fig. 5d and Supplementary Fig. 5a).

To validate this site-specific modification, we immunoprecipitated PC from control and PHD3-silenced cells and found that loss of PHD3 markedly reduced PC hydroxylation (Fig. 5e). Supporting this, MS identified a +15.9949 Da shift at P190 indicating hydroxylation (Supplementary Fig. 5b), and this was the sole residue with a clear PHD3-dependent signal (Fig. 5f and Supplementary Fig. 5c).

Next, we investigated the functional consequence of PC hydroxylation by PHD3. To this end, we hypothesized that PHD3 may suppress PC activity through this post-translational hydroxylation. To test this hypothesis, we assessed PC enzymatic activity in HEK293T cells cultured in serum-free, pyruvate-supplemented media. PC overexpression increased catalytic activity, consistent with its metabolic role, but co-expression of PHD3 abrogated this increase, restoring activity to baseline (Fig. 5g). Western blotting confirmed that total protein levels of PC and PHD3 were unaffected (Supplementary Fig. 5d,e), whereas hydroxylation of PC was increased in the presence of PHD3 (Fig. 5h), indicating post-translational inhibition via proline hydroxylation.

Together, these findings define PC as a bona fide substrate of mitochondrial PHD3 and establish site-specific hydroxylation at P190 as a direct mechanism of enzymatic suppression.

### PHD3 inhibits PC-dependent anaplerosis to restrict TCA cycle flexibility in ccRCC

**Fig. 6:**
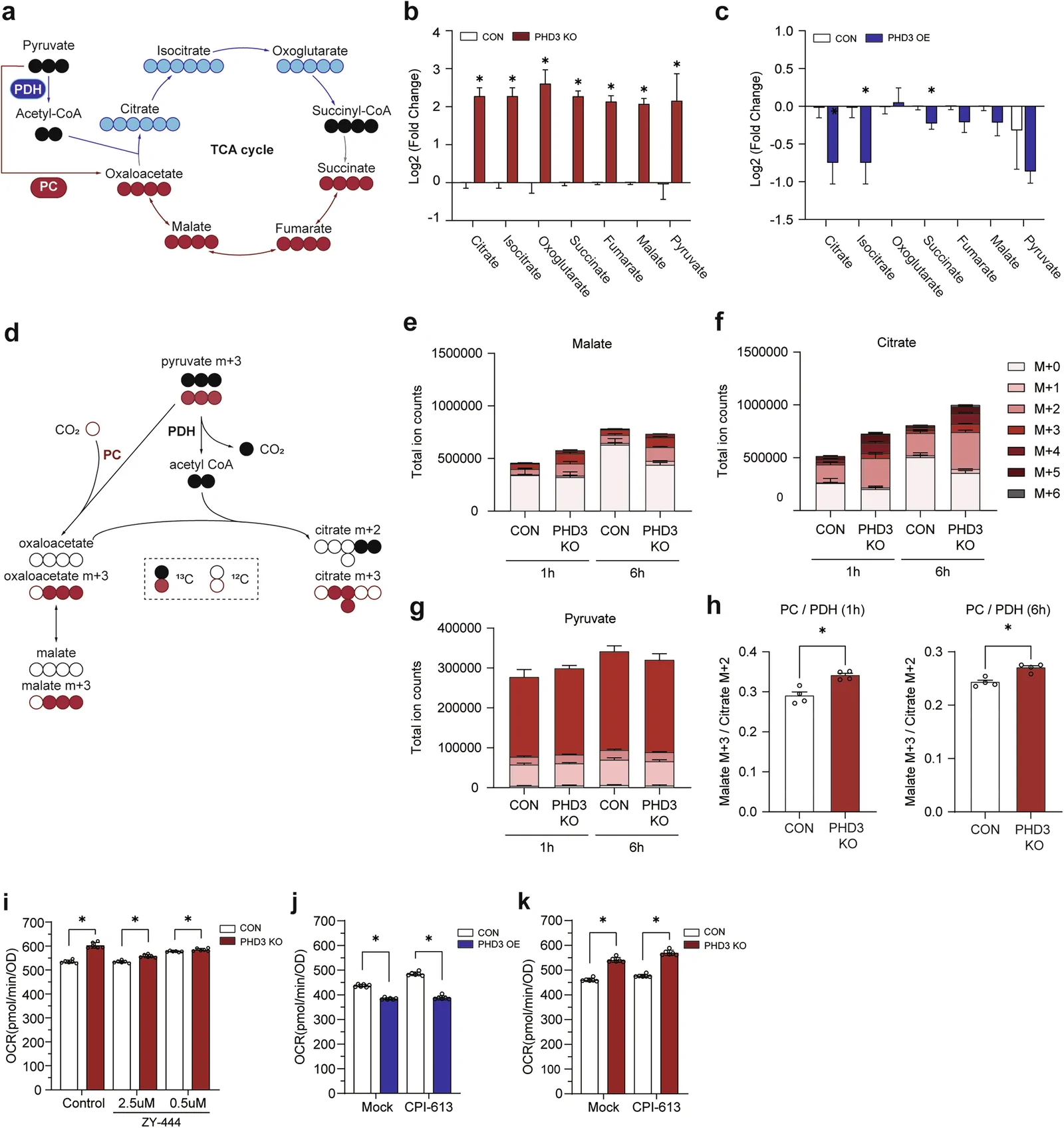
PHD3 restricts PC-dependent anaplerotic flux and metabolic flexibility in ccRCC. **a** Schematic diagram of the TCA cycle showing PC-dependent metabolites in red and PDH-dependent metabolites in blue. **b** LC–MS analysis of citrate, isocitrate, α-ketoglutarate, succinate, fumarate, and malate levels in 786-O cells with PHD3 knockout compared with control cells, assessing PC activity. **c** LC–MS analysis of the same TCA cycle intermediates in 786-O cells overexpressing PHD3 compared with control cells, evaluating the effect of PHD3 on PC activity. **d** Schematic illustrating isotopic labeling of citrate from [U-¹³C]glucose via PDH and PC during the first turn of the TCA cycle. **e** Total ion counts of malate isotopologues. **f** Total ion counts of citrate isotopologues. **g** Total ion counts of pyruvate isotopologues. **h** PC/PDH activity ratio indicating the relative contribution of PC versus PDH. 786-O cells were incubated in DMEM containing 1 mM [¹³C_3_]pyruvate for 1 h or 6 h. The citrate *m* + 2/pyruvate *m* + 3 ratio was used as a surrogate marker for PDH activity, whereas the malate *m* + 3/pyruvate *m* + 3 ratio served as a surrogate for PC activity. **i** Oxygen consumption rate (OCR) in 786-O cells treated with the PC inhibitor ZY-444 under PHD3 knockout conditions. OCR in 786-O cells treated with the PDH inhibitor CPI-613 under PHD3 overexpression (part **j**) or knockout (part **k**) conditions. ccRCC, clear cell renal cell carcinoma; KO, knockout; LC, liquid chromatography; MS, mass spectrometry; PC, pyruvate carboxylase; PDH, pyruvate dehydrogenase; PHD3, prolyl hydroxylase domain protein 3; TCA, tricarboxylic acid.

To assess how PHD3 affects global TCA cycle metabolism, we performed targeted metabolomics in 786-O renal cancer cells lacking PHD3 (Fig. 6a). PHD3 KO cells exhibited elevated levels of key TCA intermediates, including succinate, malate, and oxaloacetate (Fig. 6b and Supplementary Fig. 6a). These metabolites are frequently elevated in tumors and are associated with oncogenic signaling. By contrast, PHD3 overexpression suppressed TCA cycle metabolites, particularly those downstream of citrate (Fig. 6c and Supplementary Fig. 6b), consistent with reduced PC-mediated anaplerosis and a shift toward pyruvate dehydrogenase (PDH)-driven carbon entry.

This trend was first confirmed in murine renal cancer cells (Renca), in which PHD3 KO significantly increased PC enzymatic activity (Supplementary Fig. 6c), consistent with a functional role for PHD3 in suppressing PC.

To examine whether PHD3 loss alters broader metabolic programs, we conducted untargeted metabolomics and observed global shifts in metabolite levels. Volcano plot analysis revealed substantial changes in the metabolic landscape of PHD3-deficient 786-O cells (Supplementary Fig. 6d), whereas the reciprocal changes were observed upon PHD3 overexpression (Supplementary Fig. 6e), confirming that PHD3 orchestrates large-scale metabolic remodeling in renal cancer cells.

We next investigated the specific metabolic pathways affected by PHD3. Pathway enrichment analysis of PHD3 KO cells revealed increased flux through amino acid metabolism, nitrogen handling, and the TCA cycle (Supplementary Fig. 6f). By contrast, PHD3 overexpression suppressed these same pathways, including branched-chain amino acid biosynthesis and glycolysis/gluconeogenesis (Supplementary Fig. 6g). These results suggest that PHD3 has a key role in constraining metabolic plasticity across multiple nutrient-responsive networks.

To directly link PHD3 to PC-dependent TCA flux, we performed [U-¹³C] pyruvate isotope tracing in PHD3 KO and control 786-O cells (Fig. 6d). At both 1 h and 6 h post-labeling, PHD3-deficient cells showed increased incorporation of ¹³C into malate and citrate (Fig. 6e, f), despite equivalent pyruvate uptake (Fig. 6g). Analysis of isotopologue distribution revealed that PC-mediated (*m* + 3) labeling of malate was significantly enriched relative to PDH-mediated (*m* + 2) citrate labeling in KO cells (Fig. 6h), confirming enhanced PC-dependent anaplerosis upon PHD3 loss. To define the functional hierarchy between PHD3 and PC, we performed OCR analyses under targeted metabolic perturbations. Inhibition of PC with ZY-444 abolished the respiration differences associated with PHD3 loss (Fig. 6i), indicating that PHD3-dependent metabolic effects require PC activity. By contrast, inhibition of PDH with CPI-613 unmasked a PHD3-dependent shift in mitochondrial respiration (Fig. 6j, k), demonstrating that PC-driven anaplerosis becomes dominant when PDH flux is restricted.

Together, these findings establish PHD3 as an endogenous suppressor of PC activity. By hydroxylating and inhibiting PC, PHD3 imposes a metabolic bottleneck that restricts anaplerotic flux into the TCA cycle. This suppression limits the ability of ccRCC cells to adapt to nutrient stress and provides a mechanistic explanation for the tumor-suppressive effects of PHD3 observed in earlier experiments. The PHD3–PC regulatory axis thus emerges as a key metabolic vulnerability in renal cancer.

### PHD3 suppresses ccRCC proliferation and migration by inhibiting PC-mediated metabolic plasticity

**Fig. 7:**
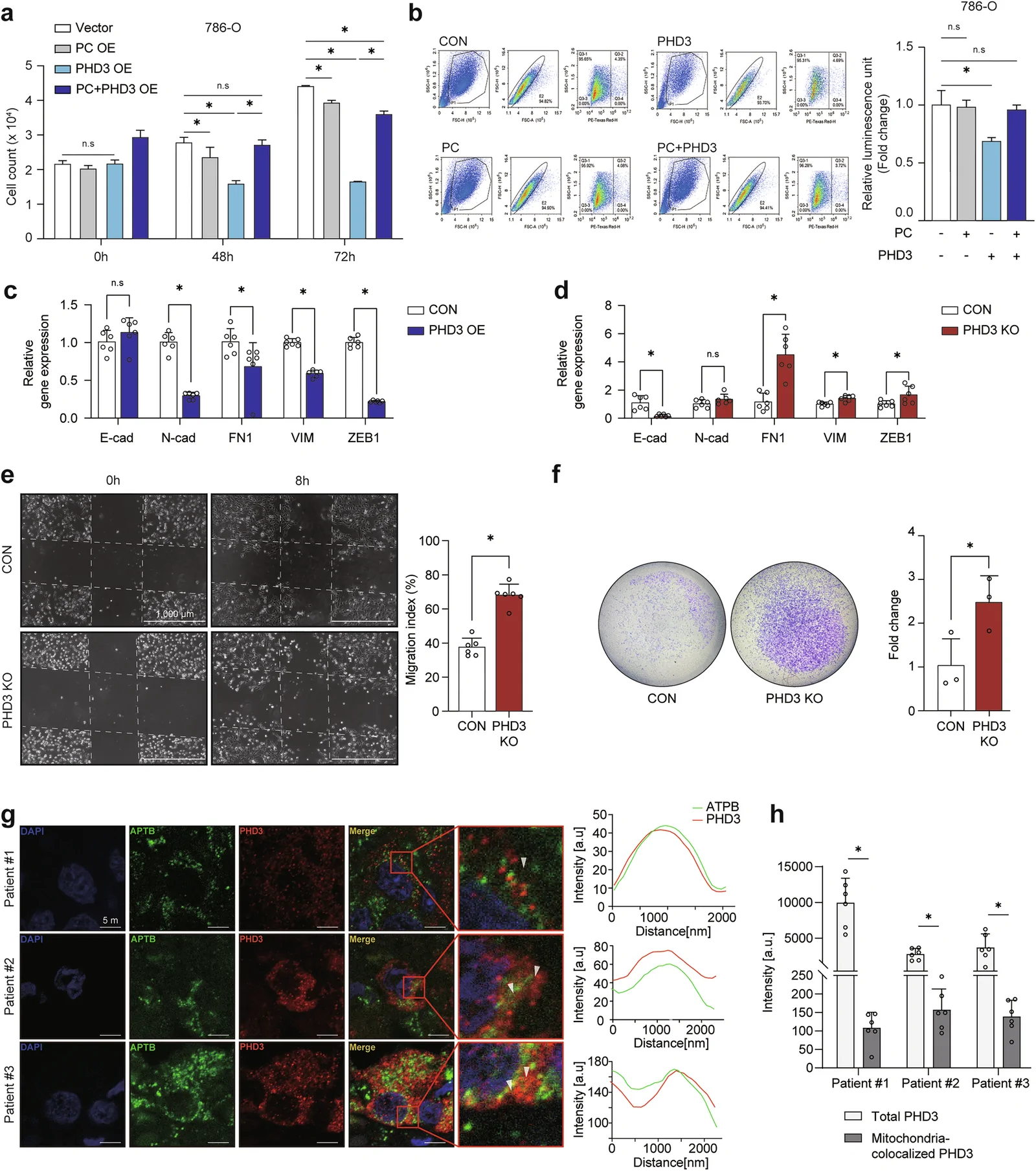
PHD3 suppresses ccRCC proliferation and migration through metabolic inhibition of PC. **a** Cell proliferation assay in 786-O cells following overexpression of PC and PHD3. **b** Quantification of cell death in 786-O cells 48 h after PC and PHD3 overexpression. Quantitative real-time PCR analysis of the indicated genes in 786-O cells with PHD3 overexpression (part **c**) or knockout (part **d**). **e** Wound healing assay in 786-O cells with PHD3 knockout compared with control cells (left), and quantification of the migration index based on the scratched region from independent experiments (right). Scale bar, 1000 μm. **f** Transwell migration assay in 786-O cells with PHD3 knockout compared with control cells (left), and quantification of crystal violet-stained cells from independent experiments (right). **g** Representative immunofluorescence staining of paraffin-embedded metastatic ccRCC patient samples showing PHD3 (red), the mitochondrial marker ATPB (green), and nuclear counterstaining with DAPI (blue), with areas of signal overlap between PHD3 and ATPB. Quantification of red–green overlap is shown in the right panel. Scale bar, 5 μm. **h** Quantitative comparison of total cellular PHD3 intensity with the mitochondria-colocalized fraction. a.u., arbitrary unit; ccRCC, clear cell renal cell carcinoma; DAPI, 4′,6-diamidino-2-phenylindole; E-cad, E-cadherin; FN-1, fibronectin-1; KO, knockout; N-cad, N-cadherin; ns, not significant; PC, pyruvate carboxylase; PHD3, prolyl hydroxylase domain protein 3; TCA, tricarboxylic acid; VIM, Vimentin.

Given that PHD3 restricts metabolic flexibility by hydroxylating mitochondrial PC, we asked whether this regulation could suppress tumor cell proliferation in ccRCC. To address this, we overexpressed PHD3 in 786-O cells, a prototypical ccRCC cell line. PHD3 expression significantly impaired cell proliferation, whereas knockdown or KO led to increased growth (Fig. 7a and Supplementary Fig. 7a, b). Co-expression of PC fully rescued the anti-proliferative effect of PHD3, highlighting a functional antagonism between the two enzymes (Fig. 7a).

Beyond growth inhibition, PHD3 overexpression induced cell death specifically in 786-O cells (Fig. 7b), whereas HEK293T cells remained unaffected (Supplementary Fig. 7c, d), highlighting a cancer-selective vulnerability. These results support a model in which PHD3 disrupts mitochondrial metabolic homeostasis, impairing cellular viability under nutrient-limiting conditions.

We next investigated whether the PHD3–PC axis influences ccRCC cell plasticity. In 786-O cells, PHD3 overexpression led to reduced expression of mesenchymal markers (N-cadherin (N-cad), Fibronectin-1 (FN1), Vimentin (VIM), ZEB1) and increased E-cadherin (E-cad) levels, indicative of EMT suppression (Fig. 7c). By contrast, PHD3 KO upregulated mesenchymal gene expression and reduced E-cadherin (Fig. 7d). Notably, PHD3 manipulation had no effect on EMT markers in HEK293T cells, further underscoring the tumor specificity of this phenotype (Supplementary Fig. 7e). Consistent with this, gene expression analysis confirmed that modulation of the PHD3–PC axis directly alters metabolic and EMT-associated transcriptional programs in 786-O cells (Fig. 7c, d and Supplementary Fig. 7f, g), indicating that these effects are selectively engaged in malignant contexts.

Functionally, loss of PHD3 markedly increased migratory potential in 786-O cells, as demonstrated by both wound-healing and transwell migration assays (Fig. 7e, f). These findings suggest that PHD3 not only limits tumor proliferation but also curbs metastatic capacity by constraining metabolic flexibility.

IF staining of metastatic ccRCC patient tissues revealed partial spatial overlap between PHD3 and the mitochondrial marker ATPB (Fig. 7g). Quantitative analysis confirmed that only a small fraction of total PHD3 localized to mitochondria across metastatic ccRCC specimens (Fig. 7h). To investigate whether this limited mitochondrial localization was associated with tumor hypoxia, we examined PHD3 localization across hypoxia gradients using CA9. Mitochondrial colocalization of PHD3 was significantly reduced in CA9-high regions compared with CA9-low regions (Supplementary Fig. 7i), indicating oxygen-dependent regulation of PHD3 localization in human tumors. Although focal regions of mitochondrial colocalization were observed, PHD3 staining was predominantly cytosolic in many tumor cells. The extent of colocalization varied across tumor cells and regions. The analyzed FFPE specimens were derived from nephrectomy samples spanning multiple pathological stages (T2a-4 ccRCC, grades 2–4) and were validated by H&E and IHC staining (HIF-1α and E-cadherin) (Supplementary Fig. 7h).

Supporting this observation, survival analysis demonstrated that among patients with high PC expression, those with higher PHD3 expression exhibited improved overall survival (Supplementary Fig. 7j). These data indicate an inverse association between PHD3 expression and the adverse clinical impact of elevated PC in human ccRCC.

**Fig. 8:**
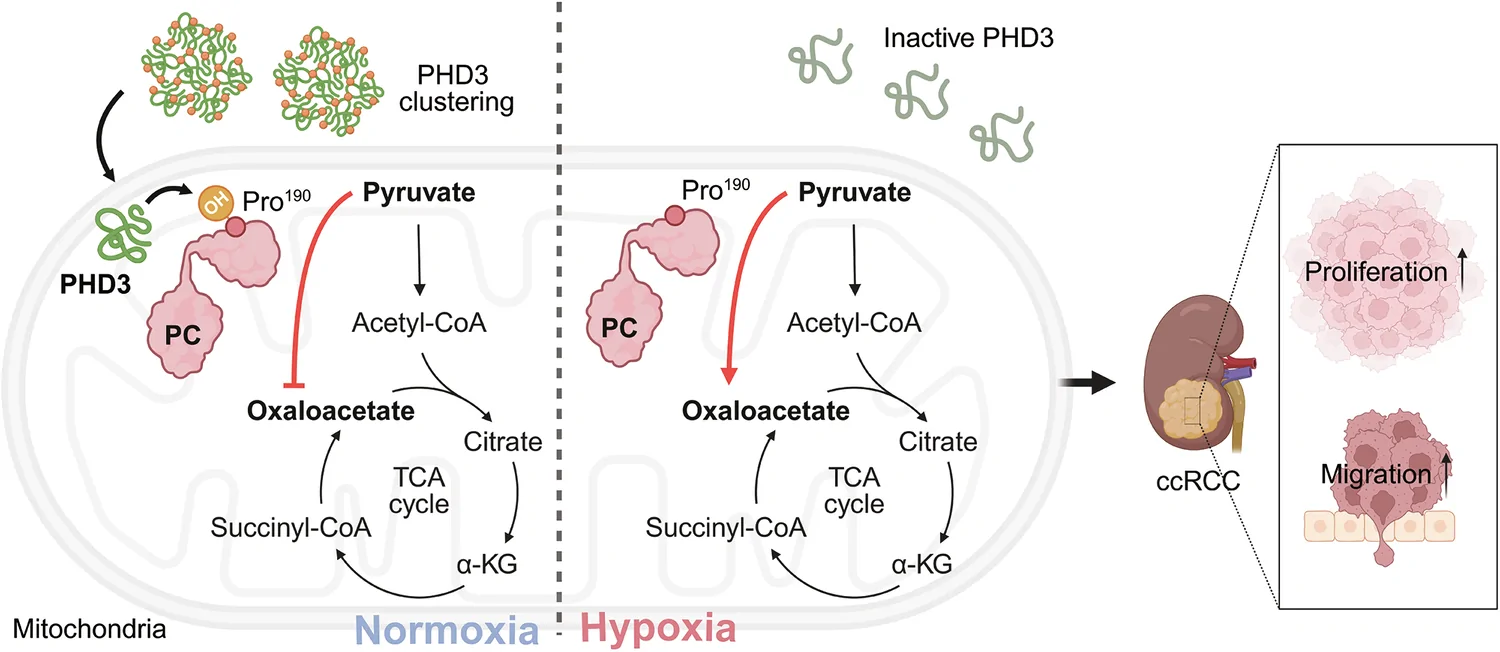
Oxygen-dependent subcellular re-localization of PHD3 links TME oxygen availability to PC regulation and ccRCC metabolic progression. Model illustrates how the oxygen-dependent intracellular distribution of PHD3 condensates formation regulates PC activity and ccRCC progression. Under normoxia, PHD3 undergoes clustering and intracellular distribution to the mitochondria, leading to hydroxylation-mediated inhibition of PC, suppressing the anaplerotic pathway. Under hypoxia, this mitochondrial distribution is decreased, PC is not hydroxylated, leading to enhanced PC-mediated anaplerotic flux, which promotes ccRCC proliferation and migration. ccRCC, clear cell renal cell carcinoma; PC, pyruvate carboxylase; PHD3, prolyl hydroxylase domain protein 3; TCA, tricarboxylic acid; TME, tumor microenvironment.

Taken together, these data support a model in which PHD3 undergoes intracellular clustering under normoxic conditions, which coincides with its mitochondrial localization and functional interaction with PC, whereas hypoxic conditions are associated with altered PHD3 behavior and increased PC-dependent metabolic activity in ccRCC tumors (Fig. 8).

## Discussion

Profound metabolic dysregulation, a hallmark of ccRCC, underlies its aggressive behavior and poor response to current therapies. Often driven by VHL inactivation, this metabolic reprogramming poses a major therapeutic challenge. Our study uncovers a previously unrecognized mechanistic link between PHD3 and PC, two critical regulators of tumor metabolism, providing a foundation for therapeutic strategies targeting this vulnerability in ccRCC. During this process, we uncovered a very unexpected phenomenon, mitochondrial import of PHD3 accompanied by dynamic subcellular redistribution. Although PHD3 has traditionally been viewed as a cytosolic enzyme regulating HIF-1α stability^29^, our study provides direct molecular evidence that PHD3 is dually localized to both the cytosol and mitochondrial matrix (Figs. 3b, c and 4e, g). This oxygen-sensitive phase behavior was unexpected, particularly as PHD3 lacks conventional IDRs typically known to mediate condensates formation, unlike PHD1 and PHD2, which possess extensive IDRs but do not exhibit punctate localization (Supplementary Fig. 4a,b). This suggests that PHD3 clustering is likely induced or modulated by interaction with other cellular factors^26^ (Fig. 4a–d), serving as a regulatory switch linking oxygen sensing to spatial control of enzymatic activity.

Furthermore, because PHD3 likely undergoes clustering via an IDR-independent pathway, the precise structural interfaces driving this process, as well as the specific transport machinery facilitating its translocation across the mitochondrial membranes, warrant further investigation in future studies.

Interestingly, a recent study in chloroplasts has suggested that protein condensation may be associated with protein translocation^30^. Given the evolutionary and structural similarities between mitochondria and chloroplasts, both double membrane organelles relying on signal -mediated protein import, this is consistent with the idea that cytosolic condensation contributes to protein targeting or import processes in such organelles.

Although the precise temporal sequence between the initiation of PHD3 intracellular clustering and its entry into the mitochondrial matrix remains to be fully resolved, our data establish a robust oxygen-dependent coupling between these two events. The finding that dynamic wild-type PHD3 undergoes import while less dynamic mutants show reduced mitochondrial localization supports a model in which the physical state of the protein is a critical gatekeeper for its subcellular trafficking and subsequent metabolic function (Fig. 4d).

Although PHD3 clustering is a fundamental biochemical process primarily governed by its local concentration, its functional coupling with mitochondrial import appears to be a hallmark of the unique pathological landscape of ccRCC. The combination of aberrant *EGLN3* upregulation and the chronically hypoxic microenvironment in ccRCC creates a primed state for this mitochondrial regulatory axis. Our comparative analyses suggest that this mechanism is specifically enriched in the ccRCC subtype, whereas it remains largely inactive in non-malignant renal cells or other RCC subtypes with lower *EGLN3* expression levels (Fig. 2a–c).

Importantly, our findings position PHD3 as a central mediator that couples TME-dependent hypoxia and normoxia states to mitochondrial metabolic regulation in ccRCC and reveal that association between PHD3 subcellular re-localization and mitochondrial import is a key mechanism for this oxygen-to-metabolism communication.

The translocated PHD3 now opens up a whole new possibility of directly interacting with its putative substrates, including BC-domain-containing proteins, within the mitochondrial matrix. Although our prior work identified ACC2, a mitochondrial outer membrane-localized biotin-dependent carboxylase, as a prominent PHD3 substrate^17^, the direct interaction and hydroxylation of mitochondrial matrix-resident proteins, such as PC, had been unclear owing to compartmental separation. Our study reveals that PHD3 directly hydroxylates PC (Fig. 5a–f), inhibiting its enzymatic activity and restricting anaplerotic flux into the TCA cycle (Fig. 6a–f), thereby limiting ccRCC cell proliferation and metastasis (Fig. 7a–f). This positions PHD3 as a key regulator of mitochondrial metabolic control.

Furthermore, we found that the hydroxylation efficiency of the substrates is not uniform across substrates. MD simulation suggests the existence of substrate preference, in which ACC2 undergoes robust hydroxylation whereas PC exhibits reduced efficiency, likely due to structural constraints or limited accessibility (Fig. 5a, b). This suggests that enzymatic access, substrate conformation, and protein dynamics contribute to substrate specificity.

This dual localization and distinct substrate specificities distinguish PHD3 from other prolyl hydroxylases such as PHD1 and PHD2. Although PHD1 and PHD2 primarily modulate tumorigenesis through regulation of the HIF response^31–33^, PHD3 uniquely suppresses cancer metabolism through a direct influence on mitochondrial enzymatic activity. This is further supported by PHD3 expression patterns in TCGA-KIRC dataset diverging from PHD1 and PHD2. Moreover, our findings indicate that PHD3 tumor-suppressive effects in ccRCC operate independently of the VHL HIF pathway (Fig. 7a, b and Supplementary Fig. 7e). Although VHL loss induces PHD3 transcription via HIF stabilization, PHD3 overexpression in VHL-deficient ccRCC cells still leads to growth suppression and apoptosis^9^. This highlights a non-redundant and direct role for PHD3 in cancer metabolism, reinforcing its biological relevance and therapeutic potential in both VHL-proficient and VHL-deficient ccRCC tumors. Consistent with this in vivo relevance, IF analysis of tumors from patients with metastatic ccRCC showed areas of spatial overlap between endogenous PHD3 and the mitochondrial marker ATPB (Fig. 7g).

Although fixed clinical samples cannot capture real-time clustering dynamics, our quantitative analysis reveals that mitochondrial colocalization of PHD3 is significantly reduced in CA9-high (hypoxic) regions compared with CA9-low regions (Supplementary Fig. 7i), providing direct evidence that PHD3 localization is dynamically regulated by spatial hypoxia gradients in human tumors. These patterns indicate that PHD3 is capable of associating with mitochondrial regions in human tumors, supporting the physiological feasibility of the mechanism we describe. Our comparative analysis of TCGA datasets and various renal cell lines suggests that the PHD3–PC regulatory axis is a specialized feature of ccRCC pathophysiology. Although the biochemical process of PHD3 clustering is fundamentally governed by protein concentration, its functional coupling with mitochondrial import appears to be a ‘perfect storm’ unique to ccRCC, in which extreme *EGLN3* upregulation and a chronically hypoxic microenvironment create the necessary prerequisites for this metabolic checkpoint.

Notably, the metabolic impact of the PHD3–PC axis appears to be highly context-dependent, targeting a specific vulnerability in malignant cells. The observation that PC inhibition triggers MET (mesenchymal-to-epithelial transition) and growth arrest in VHL-deficient ccRCC cells, but not in non-malignant, metabolically robust HEK293T cells, highlights a significant therapeutic window. This dissociation suggests that the metabolic stress induced by PHD3-mediated PC inhibition only engages EMT-related transcriptional programs in cells already primed by oncogenic metabolic addiction, rather than through a generic stress response.

We also observed an inverse correlation between PHD3 and the expression of its carboxylase substrates at the transcriptomic level (Fig. 2g, h), suggesting a potential feedback mechanism influencing gene expression.

In sum, this work provides a fundamental basis of PHD3-mediated mitochondrial metabolic regulation with direct clinical relevance. By identifying a non-canonical function of PHD3 in the mitochondrial matrix where it suppresses cancer cell metabolism through PC hydroxylation and inhibition, our study uncovers an unexpected dimension of oxygen sensing that links phase separation, mitochondrial targeting, and metabolic control. Collectively, these findings suggest PHD3 as a master integrator that links TME-derived oxygen cues to mitochondrial metabolic outputs through its oxygen-dependent subcellular re-localization. These findings highlight the therapeutic potential of the PHD3–PC axis as a metabolic vulnerability in kidney cancer. Further exploration of PHD3 substrates^34–36^, its dynamic clustering behavior, and mitochondrial localization mechanisms in vivo may uncover new strategies to target metabolic vulnerabilities in cancer^37^.

## Supplementary information

Supplementary Information
