# Supplementary material for: Oxygen-dependent subcellular redistribution of PHD3 links the hypoxic microenvironment to mitochondrial metabolic reprogramming in ccRCC
Source: Exp Mol Med. 2026 Sep 2;58(9):2903–18. doi: 10.1038/s12276-026-01814-z (PMC13633246; doi:10.1038/s12276-026-01814-z)
Supplement: Supplementary file 1 [file 12276_2026_1814_MOESM1_ESM.pdf]

Supplementary Figure 1

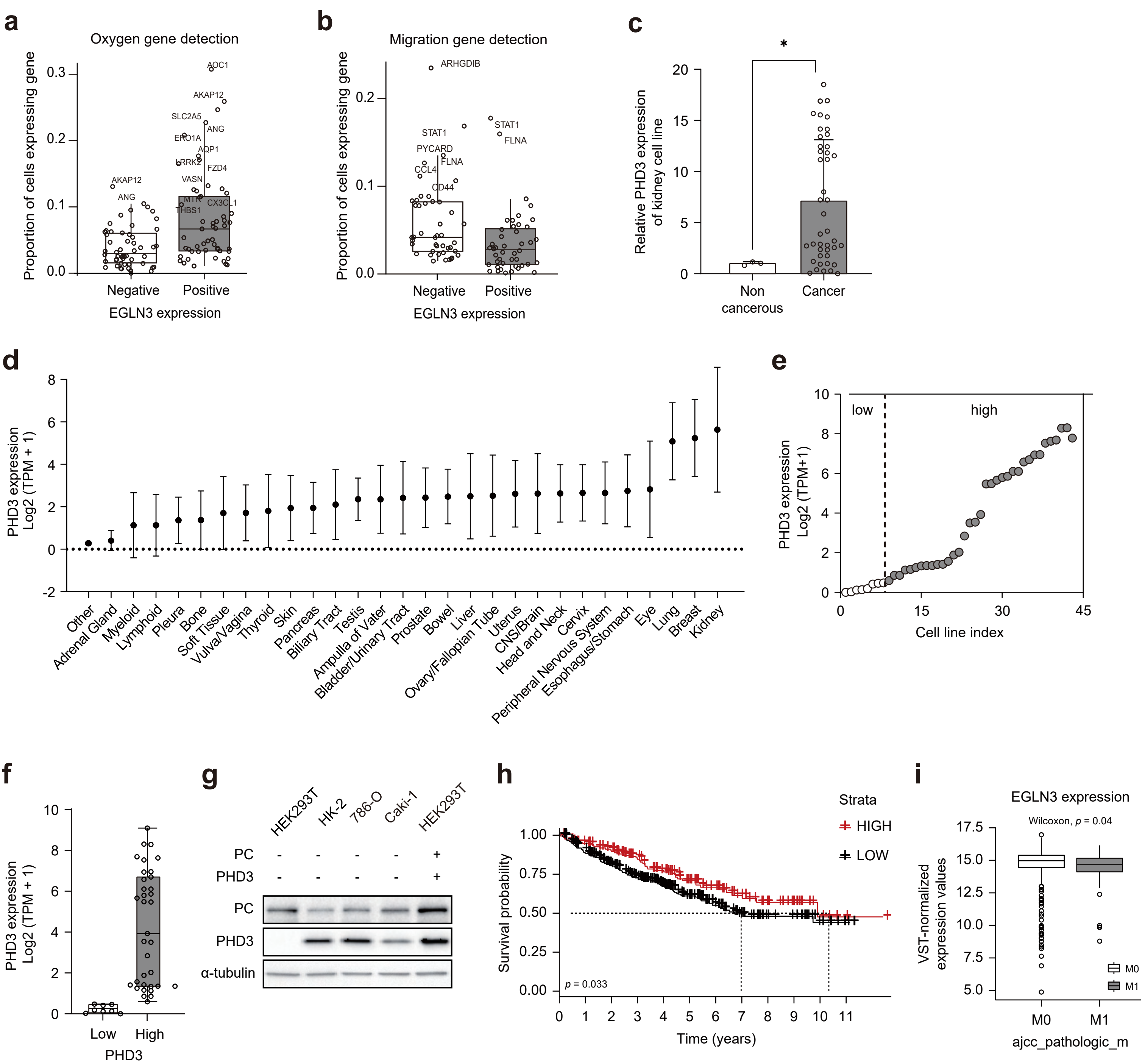

Supplement figure 1. PHD3 expression is associated with suppressed metastatic programs in ccRCC. (a-b) Box plots showing the proportion of cells expressing marker genes related to oxygen sensing (a) or cell migration/EMT (b), grouped by *EGLN3* expression status (*EGLN3* + in gray, *EGLN3* − in white). (c) Comparison of PHD3 expression between normal kidney and kidney cancer cell lines from DepMap. (d) PHD3 gene expression across various cancer cell lines, based on data from the DepMap database. (e) Stratification of kidney cancer cell lines into low and high PHD3 expression groups, based on the average expression level in normal kidney cell lines. Approximately 80% of cancer cell lines exhibited high PHD3 expression. (f) Detailed expression profiles of PHD3 across individual kidney cancer cell lines in DepMap, grouped as high or low relative to normal. (g) Western blot analysis of PHD3 expression in kidney cell lines (HK-2, 786-O, and Caki-1) and in HEK293T cells overexpressing the indicated proteins. (h) Kaplan–Meier survival curves showing that high *EGLN3* expression is associated with longer overall survival in TCGA-KIRC patients. (i) Box plot of variance-stabilized *EGLN3* expression stratified by AJCC pathologic metastasis status (M0: no metastasis; M1: distant metastasis) in TCGA-KIRC. Expression values were normalized using DESeq2’s variance-stabilizing transformation (VST); statistical significance was assessed using a Wilcoxon rank-sum test.

Supplementary Figure 2

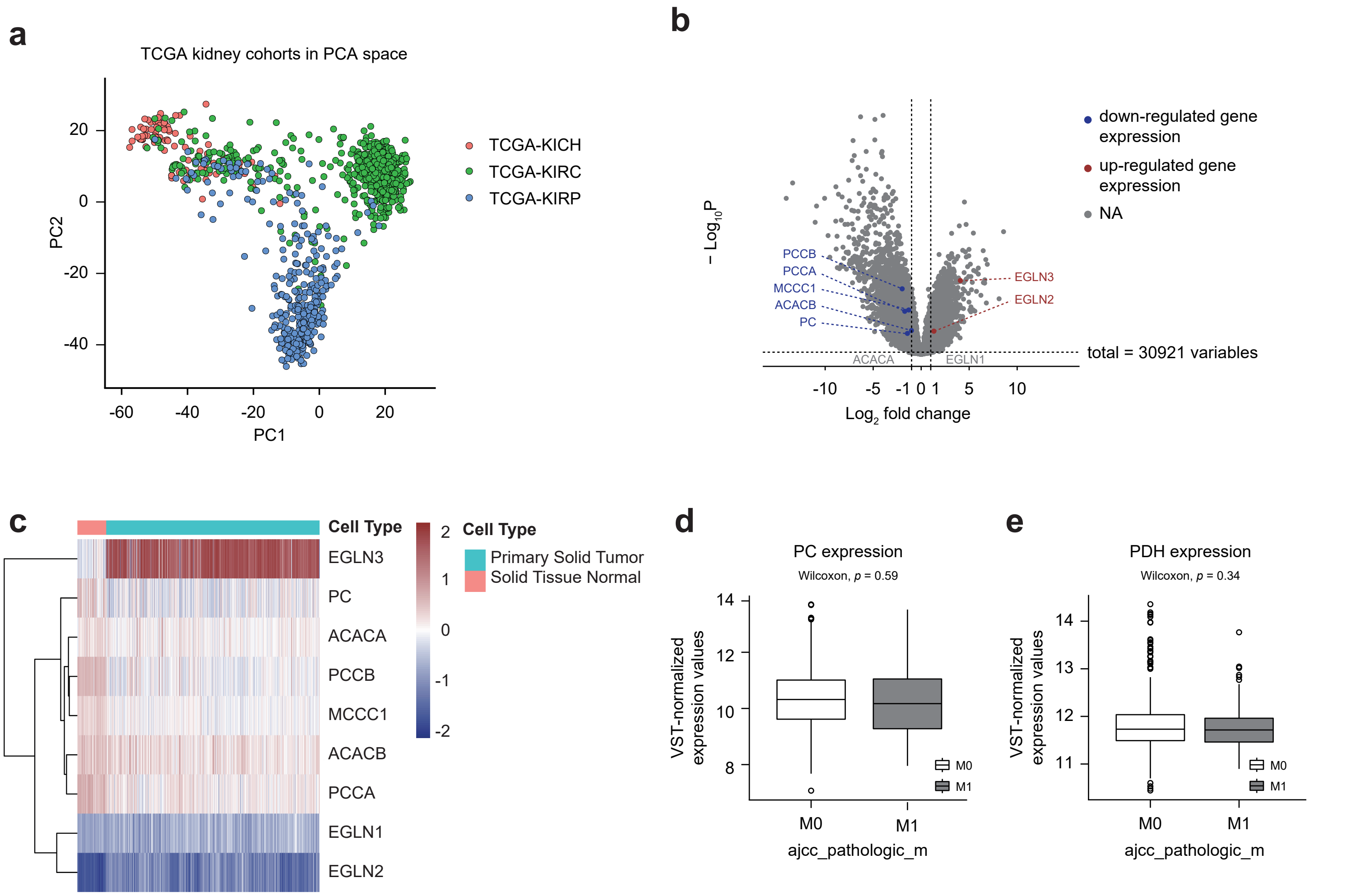

Supplement figure 2. PHD3 targets BC domain-containing mitochondrial enzymes with inverse expression correlation. (a) Principal component analysis (PCA) using highly variable genes demonstrates separation of KIRC, KIRP, and KICH samples. (b) Volcano plot showing differential expression of EGLN 1–3 and mitochondrial carboxylases (ACACA, ACACB, MCCC1, PC, PCCA, PCCB) in TCGA samples. (c) Heatmap of z-score–nxormalized expression levels of EGLN1 (PHD2), EGLN2 (PHD1), EGLN3 (PHD3), and carboxylases in TCGA-KIRC samples. Tissue type is indicated above the heatmap: solid tissue normal (pink) and primary tumor (cyan). (d-e) Box plots of variance-stabilized expression levels of PC (d) and PDH (e) stratified by metastasis status in TCGA-KIRC.

Supplementary Figure 3

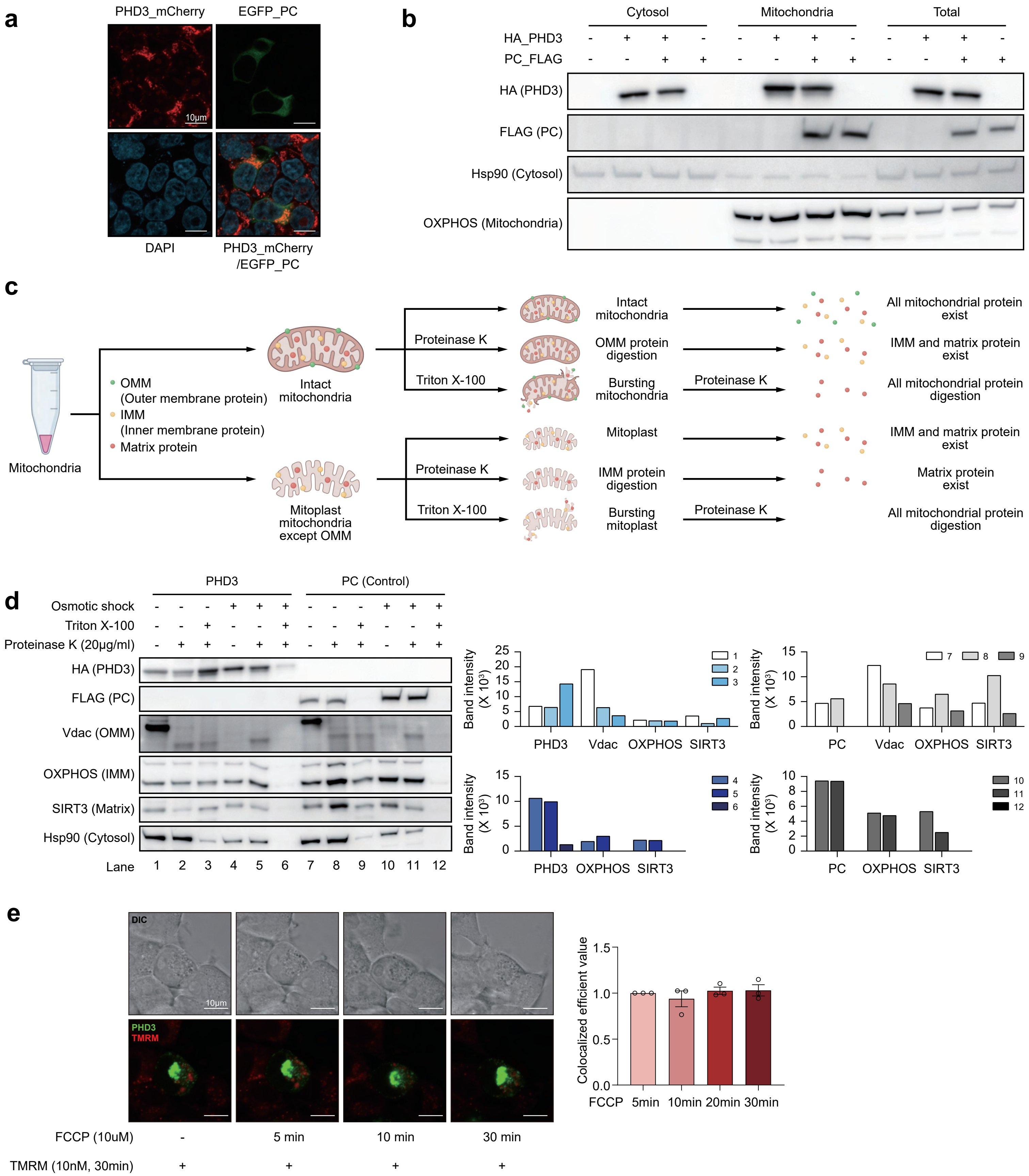

Supplement figure 3. PHD3 localizes to the mitochondrial matrix. (a) Fluorescence images showing co-localization of PHD3 and PC in HEK293T cells. PHD3 was visualized with an mCherry tag (red), and PC with a GFP tag (green). Nuclei were stained with DAPI (blue). Scale bar, 10 μm. (b) Western blot analysis of PHD3 and PC localization following crude mitochondrial fractionation in HEK293T cells. OXPHOS and Hsp90 were used as mitochondrial and cytosolic markers, respectively. (c) Schematic overview of the proteinase protection assay used to determine submitochondrial localization. (d) Western blot analysis of the proteinase protection assay for PHD3 and PC after selective rupture of the mitochondrial outer membrane (left). Proteinase K was used to digest proteins located outside the mitochondrial matrix. Quantification of western blot signals from independent experiments is shown for PHD3 (lanes 1–6, middle) and PC (lanes 7–12, right). (e) Live-cell imaging of HEK293T cells expressing PHD3–GFP (green) and stained with TMRM (red; 10 nM, 30 min) following FCCP treatment (10 uM) over the indicated time course. Scale bar, 20 μm.The accompanying graph shows time-dependent changes in TMRM–PHD3 colocalization signals.

Supplementary Figure 4

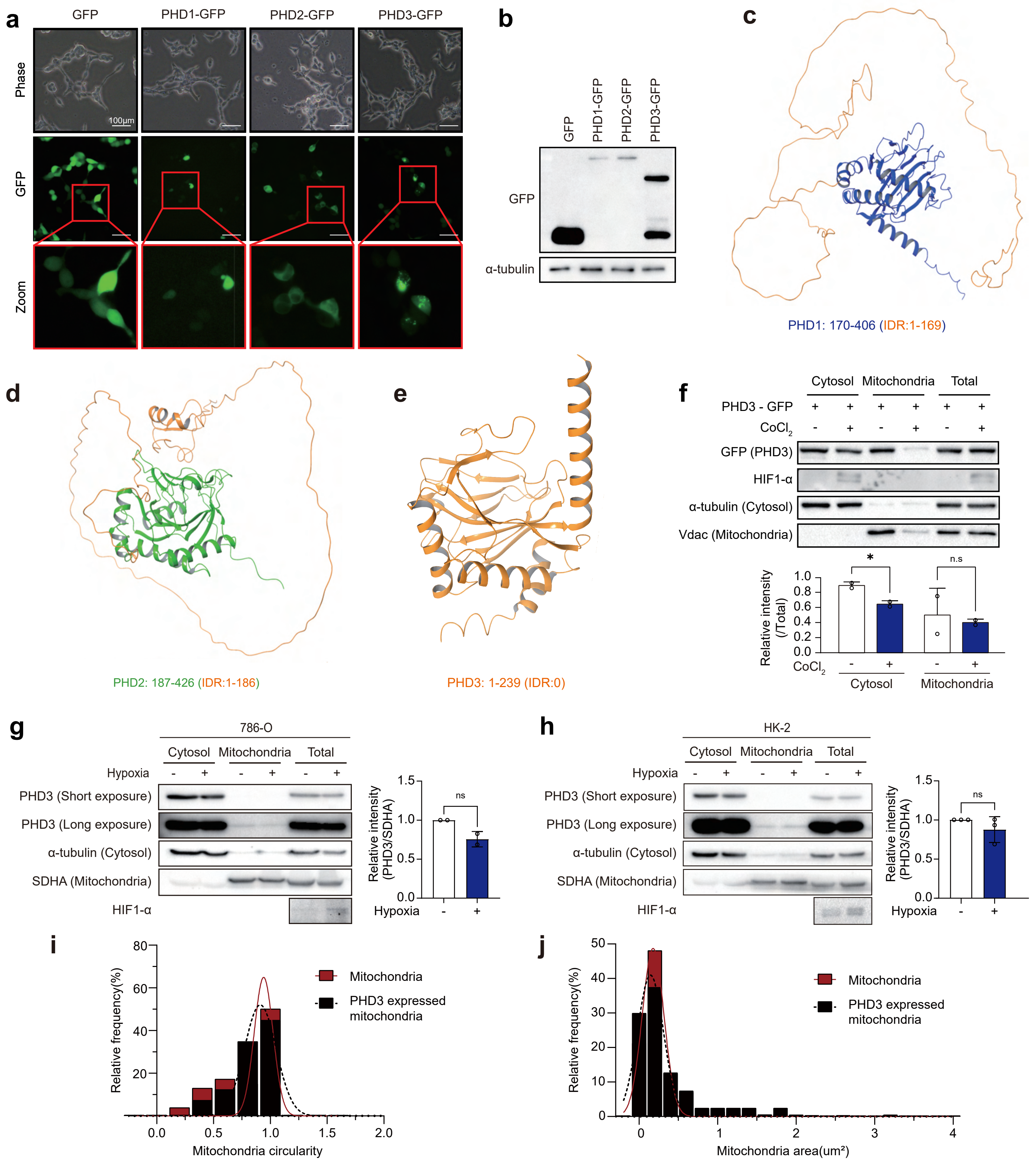

Supplement figure 4. Oxygen-sensitive intracellular distribution of PHD3 is associated with its localization between cytosol and mitochondria. (a) Representative fluorescence images showing the subcellular localization of GFP, PHD1-GFP, PHD2-GFP, and PHD3-GFP in HEK293T cells. Scale bar, 100 μm (b) Quantification of western blot signals for the indicated proteins shown in (a). (c) Predicted structure of PHD1 (residues 170–406, blue), with an intrinsically disordered region (IDR) at the N-terminus (residues 1–169, light orange). (d) Predicted structure of PHD2 (residues 187–426, green), with an N-terminal IDR (residues 1–186, light orange). (e) Predicted structure of PHD3 (residues 1–239, orange), showing minimal or no significant intrinsically disordered region (IDR). (f) Western blot analysis of PHD3 localization following crude mitochondrial fractionation in HEK293T cells treated with or without $CoCl_2$ (top). Quantification of PHD3 protein levels from independent experiments is shown (bottom). (g-h) Subcellular fractionation were performed in 786-O cells (g) and HK-2 cells (h). Representative immunoblots are shown on the left, with corresponding quantification of PHD3 protein levels from independent experiments on the right. (i-j) Distribution of mitochondrial morphology according to PHD3 localization. Histograms show mitochondrial circularity (i) and area (j) in mitochondria with or without PHD3.

Supplementary Figure 5

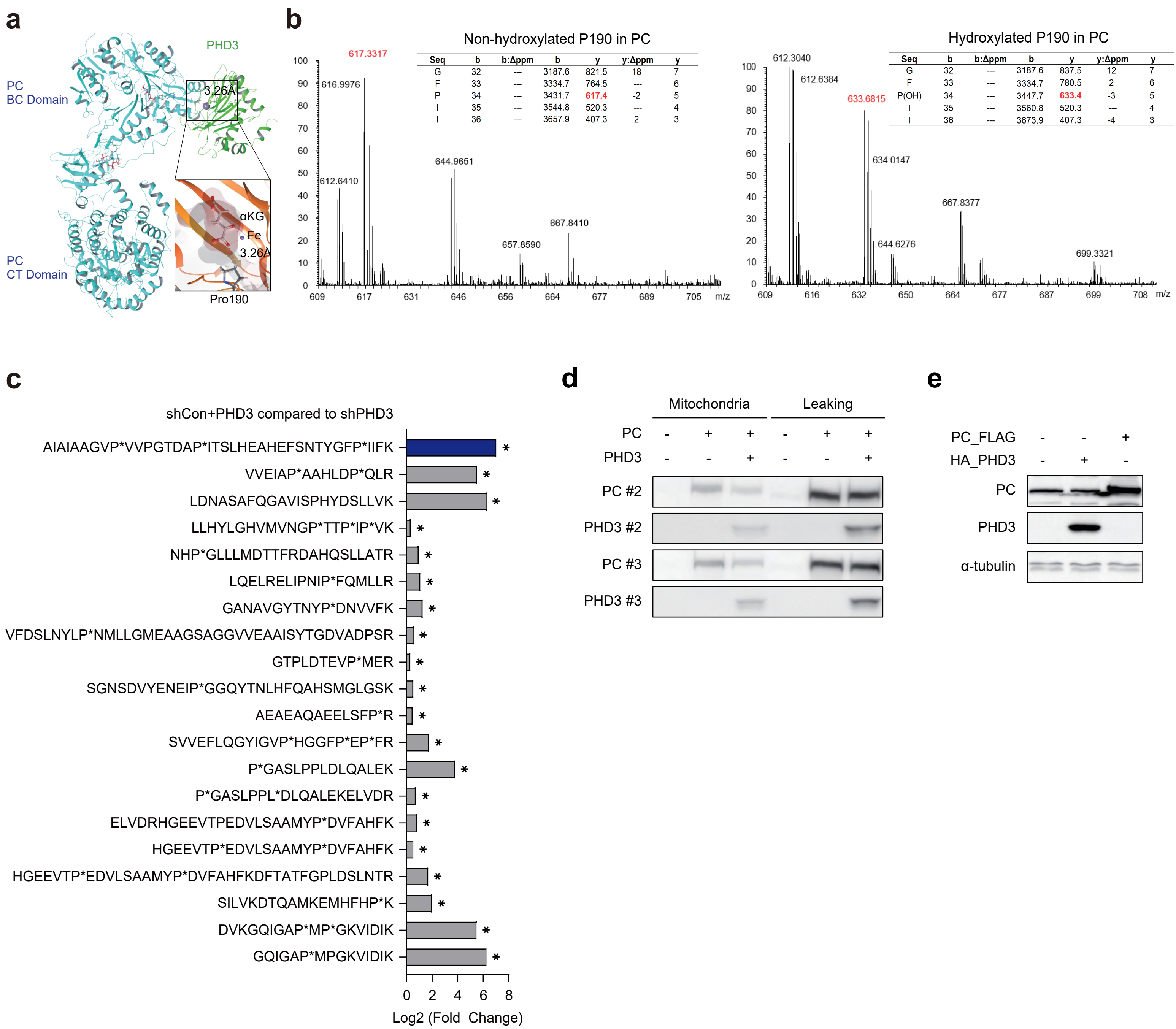

Supplement figure 5. Mitochondrial PHD3 inhibits PC activity via hydroxylation of proline 190. (a) Structural modeling of the PHD3–PC complex showing the spatial relationship between α-ketoglutarate (αKG) and the hydroxylation target, proline 190 of PC. The distance between αKG and the substrate proline is indicated in angstroms (Å). (b) Mass spectrometry analysis showing non-hydroxylated (top) and hydroxylated (bottom) forms of the proline residue at position P190 in immunoprecipitated PC. 'b' ions represent N-terminal fragments, and 'y' ions represent C-terminal fragments. (c) Relative abundance of hydroxylated proline residues in PC immunoprecipitates from HEK293T cells overexpressing or depleted of PHD3. (d) Immunoblot confirming PHD3 and PC expression for use in the PC enzymatic activity assay. (e) Immunoblot analysis showing protein levels of PHD3 and PC in cells overexpressing each construct.

Supplementary Figure 6

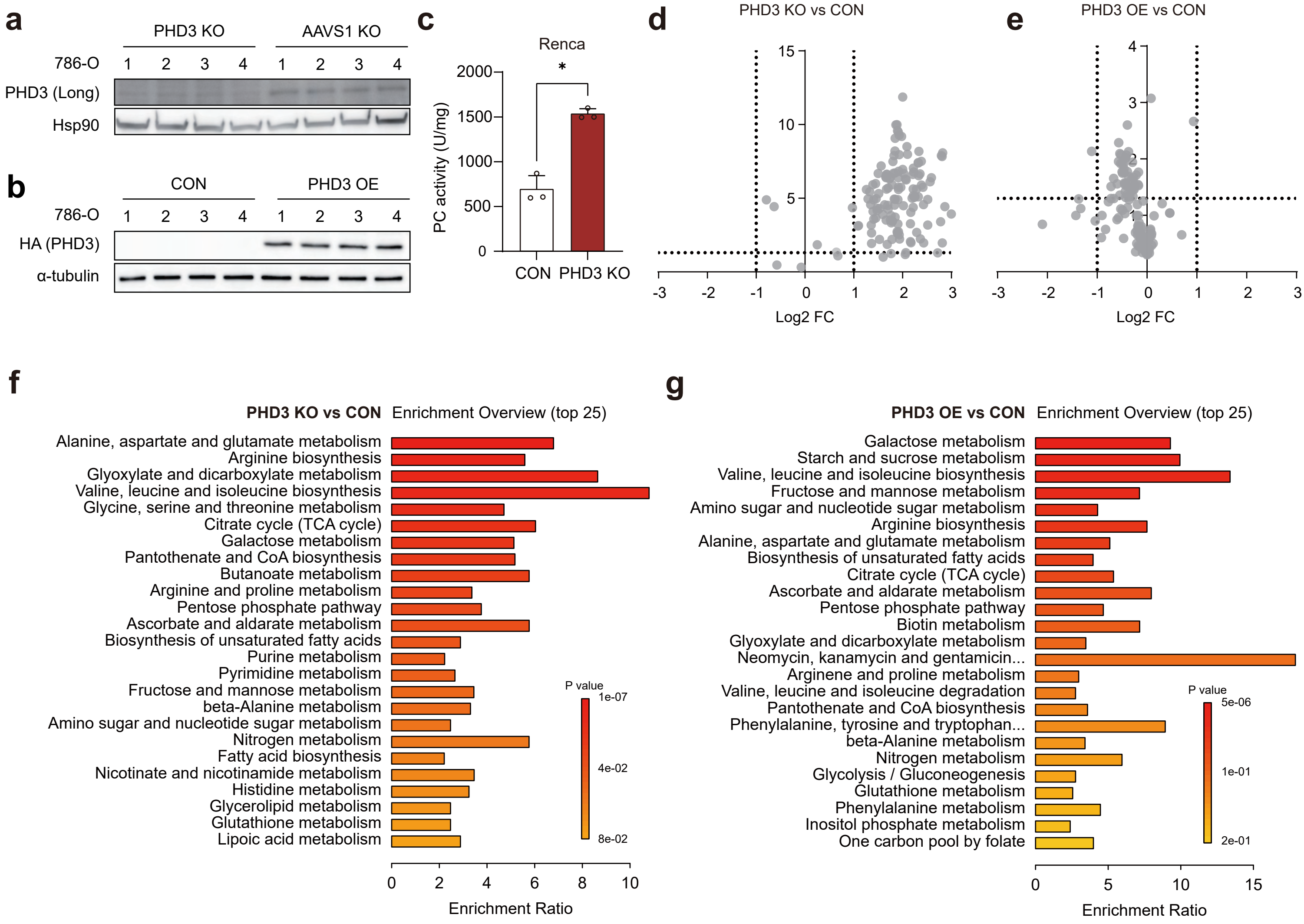

Supplement figure 6. PHD3 restricts TCA cycle flexibility by inhibiting PC-dependent anaplerosis. (a-b) Western blot validation of PHD3 expression in cells used for metabolomic analyses shown in Figure 6B (a) and Figure 6C (b). (c) Enzymatic activity of pyruvate carboxylase measured in Renca cells with or without PHD3 knockout. (d) Volcano plot showing significantly elevated metabolites in PHD3-depleted 786-O cells compared to control. (e) Volcano plot highlighting decreased metabolites in 786-O cells overexpressing PHD3. (f) Bar plot of enriched metabolic pathways based on elevated metabolites in PHD3-depleted 786-O cells. (g) Bar plot of enriched pathways among downregulated metabolites in PHD3-overexpressing 786-O cells.

Supplementary Figure 7

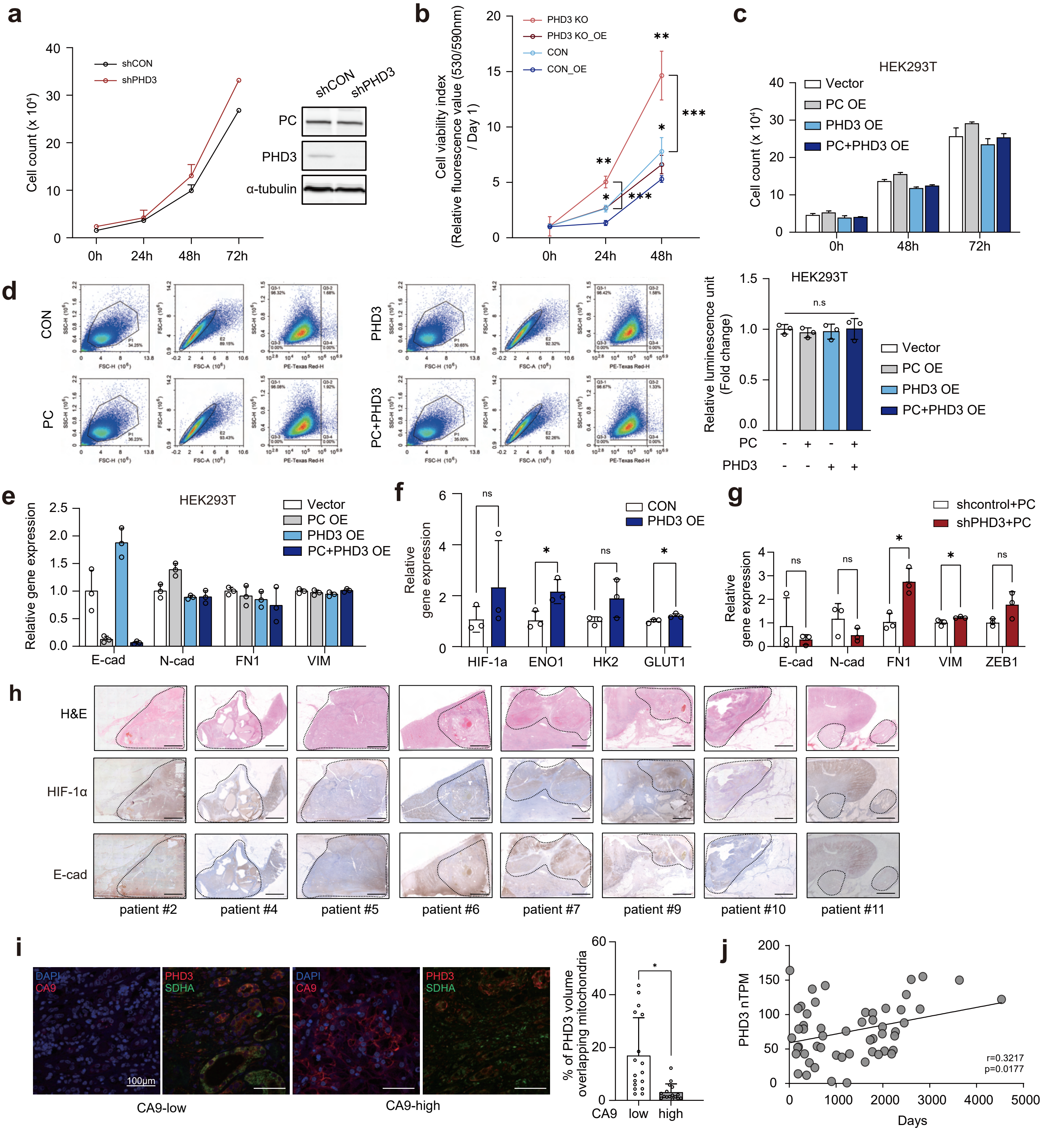

Supplement figure 7. PHD3 suppresses proliferation and migration via inhibition of PC-driven metabolic plasticity. (a) Cell proliferation assay in 786-O cells following PHD3 knockdown. (b) Resazurin-based cell proliferation assay performed in PHD3 knockout 786-O cells. (c) Propidium iodide (PI) staining–based cell viability assay in HEK293T cells overexpressing PC and PHD3. (d) Quantification of cell death by Annexin V/7-AAD staining in HEK293T cells 48 hours after co-overexpression of PC and PHD3. (e) qRT-PCR analysis of mRNA expression of the indicated genes in HEK293T cells overexpressing PC or PHD3. (f) mRNA levels of hypoxia-associated genes in 786-O cells following PHD3 overexpression. (g) mRNA levels of EMT-associated genes in 786-O cells following PHD3 knockdown with PC overexpression. (h) Representative histological images of paraffin-embedded human ccRCC tumor sections. Serial sections from each patient sample (arranged from left to right in the same order) were subjected to hematoxylin and eosin (H&E) staining, immunohistochemical staining for HIF-1α and E-cadherin. Images illustrate intratumoral heterogeneity in hypoxia signaling and epithelial marker expression across patient samples. Scale bar, 5,000 μm. (i) Representative immunofluorescence images of CA9-low and CA9-high regions in FFPE patient tissue sections (left). Serial sections from the same regions were stained for CA9 (red; DAPI, blue) or for PHD3 (red) and the mitochondrial marker SDHA (green). Quantification of PHD3 colocalization with mitochondria in these regions is shown (right). Scale bar, 100 μm. (j) Pearson correlation between PHD3 expression and overall survival in the top 10% of patients with high PC expression in TCGA-KIRC.

Supplementary Table 1. Primer sequence used in this study

Name	Application	Sequence	
Control shRNA	shRNA knockdown	Forward	CCGGCCGTCATAGCGATAACGAGTTCTCGAGAACTCGTTATCGCTATGACGGTTTTTG
		Reverse	AATTCAAAAACCGTCATAGCGATAACGAGTTCTCGAGAACTCGTTATCGCTATGACGG
shPHD3		Forward	CCGGCACCTGCATCTACTATCTGAACTCGAGTTCAGATAGTAGATGCAGGTGTTTTTG
		Reverse	AATTCAAAAACACCTGCATCTACTATCTGAACTCGAGTTCAGATAGTAGATGCAGGTG
human PHD3	CRISPR knockout	Forward	CACCGGACCAGATCACGTGGATCGG
		Reverse	AAACCCGATCCACGTGATCTGGTCC
human CON(AAVS1)		Forward	CACCGCTGGACTCCACCAACGCCGA
		Reverse	AAACTCGGCGTTGGTGGAGTCCAGC
mouse PHD3		Forward	CACCGCGCCATGTGGACAACCCCAA
		Reverse	AAACTTGGGGTTGTCCACATGGCGC
mouse CON(AAVS1)		Forward	CACCGCTCGACTCCACCAACGCGGA
		Reverse	AAACTCCGCGTTGGTGGAGTCGAGC
VIM(Vimentin)	qRT-PCR	Forward	GAGAACTTTGCCGTTGAAGC
		Reverse	GCTTCCTGTAGGTGGCAATC
FN1 (Fibronectin-1)		Forward	CAGTGGGAGACCTCGAGAAG
		Reverse	TCCCTCGGAACATCAGAAAC
N-cad (N-cadherin)		Forward	ACAGTGGCCACCTACAAAGG
		Reverse	CCGAGATGGGGTTGATAATG
E-cad (E-cadherin)		Forward	TGCCCAGAAAATGAAAAAGG
		Reverse	GTGTATGTGGCAATGCGTTC
ZEB1		Forward	AAGTGGCGGTAGATGGTAATG
		Reverse	AGGAAGACTGATGGCTGAAATAA
B2M (Beta-2 Microglobulin)		Forward	AGATGAGTATGCCTGCCGTGTGAA
		Reverse	TGCTGCTTACATGTCTCGATCCCA
PHD3 H196A	Cloning	Forward	CCGAAGTGCAGCCCTCTTACGC
		Reverse	CTGGGTTCCTACGATCTGACCAGAAGA
PHD3 R205K		Forward	AATATGCTATGACTGTCTGGTACTTTGATGCTG

		Reverse	TGGTTGCGTAAGAGGGCTGCA
PC P190A		Forward	GCCATCATCTTCAAGGCGGCCTATGG
		Reverse	GAAGCCGTAGGTGTTGGAGAACTCG
PHD1 add attB sites		Forward	GGGGACAACTTTGTACAAAAAAGTTG GCATGGACAGCCCGTGC
		Reverse	GGGGACAACTTTGTACAAGAAAGTTG GGTAGGTGGGCGTAGGCG
PHD2 add attB sites		Forward	GGGGACAACTTTGTACAAAAAAGTTG GCATGGCCAATGACAGCGGC
		Reverse	GGGGACAACTTTGTACAAGAAAGTTG GGTAGAAGACGTCTTTACCGACCG

Supplementary Table 2. Plasmids used in this study.

Plasmid Name	Source	Identifier
pcDNA3.1-ACC2-FLAG	From the laboratory of Dr. Marcia Haigis's lab	N/A
pcDNA3.1-CMV-FLAG	From the laboratory of Dr. Mikko Taipale's lab	N/A
pcDNA3.1-CMV-HA	From the laboratory of Dr. Marcia Haigis's lab	N/A
pcDNA3.1-HA-PHD3	From the laboratory of Dr. Marcia Haigis's lab	N/A
pcDNA3.1-MCCC1-FLAG	This paper	N/A
pcDNA3.1-PC WT-FLAG	This paper	N/A
pcDNA3.1-PC P190A-FLAG	This paper	N/A
pcDNA3.1-PCCA-FLAG	This paper	N/A
pcDNA3.1-PCCB-FLAG	This paper	N/A
pDEST-CMV-GFP	From the laboratory of Dr. Chan Young Park’s lab	N/A
pDEST-CMV-mCherry	From the laboratory of Dr. Chan Young Park’s lab	N/A
pDEST-PHD1-GFP	This paper	N/A
pDEST-PHD2-GFP	This paper	N/A
pDEST-PHD3 WT-GFP	This paper	N/A
pDEST-PHD3 H196A-GFP	This paper	N/A
pDEST-PHD3 R205K-GFP	This paper	N/A
pDEST-PHD3 WT-mCherry	This paper	N/A
pDONR223-MCCC1	From the laboratory of Dr. Dae-Kyum Kim's lab	N/A

pDONR223-PC	From the laboratory of Dr. Dae-Kyum Kim's lab	N/A
pDONR223-PCCA	From the laboratory of Dr. Dae-Kyum Kim's lab	N/A
pDONR223-PCCB	From the laboratory of Dr. Dae-Kyum Kim's lab	N/A
pDONR223-PHD1	This paper	N/A
pDONR223-PHD2	This paper	N/A
pDONR223-PHD3	From the laboratory of Dr. Dae-Kyum Kim's lab	N/A
pLKO.1-PHD3	This paper	N/A
pQE-PC WT-Strep-His	This paper	N/A
pQE-PC P190A-Strep-His	This paper	N/A
PX459-CON (human)	This paper	N/A
PX459-CON (mouse)	This paper	N/A
PX459-PHD3 (human)	This paper	N/A
PX459-PHD3 (mouse)	This paper	N/A

Supplementary Table 3. Antibodies used in this study

**Antibody**	**Source**	**Identifier**
Anti-Caspase-3	Cell Signaling Technologies	# 9662S
Anti-Cleaved Caspase-3 (Asp175)	Cell Signaling Technologies	# 9661S
Anti-DYKDDDDK Tag	Cell Signaling Technologies	# 2368S
Anti-GAPDH	Cell Signaling Technologies	# 2118
Anti-GFP	Cell Signaling Technologies	# 2555
Anti-HA	Cell Signaling Technologies	# 3724S
Anti-HIF1-α	Cell Signaling Technologies	# 36169S
Anti-His	Abcam	# ab18184
Anti-Hsp90	Santa Cruz Biotechnology	# F2922
Anti-hydroxyproline	ADVANCED TARGETING SYSTEMS	# AB-T044
Anti-Mouse IgG, HRP-linked	Cell Signaling Technologies	# 7076S
Anti-OXPHOS	Abcam	# ab110411
Anti-PC	Proteintech	# 16588-1-AP
Anti-PHD3	Novus Biologicals	# NB100-139
Anti-PHD3	Santa Cruz Biotechnology	# sc-46030
Anti-Rabbit IgG, HRP-linked	Cell Signaling Technologies	# 7074S
Anti-SIRT3	Santa Cruz Biotechnology	# sc-365175
Anti-Tom20	Santa Cruz Biotechnology	# sc-136211
Anti-Vdac	Cell Signaling Technologies	# 4866

Anti-α-tubulin	Santa Cruz Biotechnology	# sc-5286
Apoptosis Antibody Sampler Kit II	Cell Signaling Technologies	# 34811T

Supplementary Table 4. Reagent and materials used in this study

**Reagent/Material**	**Source**	**Identifier**
EZview anti-Flag Affinity Gel	Sigma	# F2426
EZview anti-HA Affinity Gel	Sigma	# E6779
Cobalt chloride	Sigma	# 232696
Dimethyl sulfoxide	Sigma	# D8418-100ML
Dimethyloxaloylglycine	Sigma	# D3695-10MG
Dimethyloxaloylglycine	Frontier Scientific	# 89464-63-1
Gateway BP Clonase II Enzyme mix	Invitrogen	# 11789100
Gateway LR Clonase II Enzyme mix	Invitrogen	# 11791100
Lipofectamine 2000	Invitrogen	# 11668019
Mitotracker, Orange	Invitrogen	# M7510
ProLong Gold Antifade Mountant with DNA	Invitrogen	# P36941
Revitablot™ Western Blot Stripping Buffer	ROCKLAND	# MB-085-0050
SuperSignal™ West Femto Maximum Sensitivity Substrate	ThermoFisher Scientific	# 34095
Western Lightning Pro	PerkinElmer	NEL121001EA

Supplementary Table 5. Software and tools used in this study.

**Software and Algorithms**	**Source**	**Identifier**
GraphPad Prism	GraphPad Software	N/A
ImageJ	NIH	N/A
MitoFates 1.2	Institut Pasteur	N/A
RStudio	Posit, PBC	N/A
WebLogo	University of California, Berkeley	N/A

CONTACT FOR REAGENT AND RESOURCE SHARING

Further information and requests for resources and reagents should be directed to and will be fulfilled by the Lead Contact, Haejin Yoon and Hunsang Lee (haejinyoon@unist.ac.kr, hunsang@korea.ac.kr).
